# Top-Down Proteomics of Zebrafish Brain Regions Using Capillary Zone Electrophoresis-Tandem Mass Spectrometry

**DOI:** 10.1021/acs.jproteome.6c00007

**Published:** 2026-04-17

**Authors:** Mehrdad Falamarzi Askarani, William Poulos, Maryam Rahimzadeh Dashtaki, Fei Fang, Jose B. Cibelli, Liangliang Sun

**Affiliations:** † Department of Chemistry, 3078 Michigan State University, 578 S Shaw Lane, East Lansing, Michigan 48824, United States; ‡ Department of Animal Science, 3078 Michigan State University, East Lansing, Michigan 48824, United States; § Department of Large Animal Clinical Sciences, Michigan State University, East Lansing, Michigan 48824, United States

**Keywords:** top-down proteomics, zebrafish brain, proteoform, capillary zone electrophoresis-mass spectrometry, post-translational modifications, spatial proteomics, neuropeptides, region-specific expression

## Abstract

Understanding region-specific proteoform profiles in the brain is crucial for deciphering neural function and identifying therapeutic targets. Zebrafish (Danio rerio) is a valuable vertebrate model for neuroscience research due to its substantial conservation of brain structure and function with that of mammals. We present a label-free quantitative top-down proteomics (TDP) analysis of distinct zebrafish brain regions using microdissection and capillary zone electrophoresis-tandem mass spectrometry (CZE-MS/MS). We analyzed four anatomically distinct regions—telencephalon (Tele), combined habenula-optic tectum (Tec/Hab), cerebellum (Cer), and medulla (Med)—identifying 1,050 proteoforms from 336 proteins. Only 89 proteoforms (5.1%) were shared across all regions, demonstrating substantial proteoform heterogeneity. Quantitative comparisons of proteoform intensity between any two brain regions revealed drastic proteoform abundance differences. Interestingly, proteoforms of the same genes (i.e., *sncb, calm1a, mbpa, pcp4l1, apoa2*, and *nefma*) showed opposite expression patterns between brain regions, indicating potential proteoform-specific functions. Nearly 153 neuropeptides were identified using a recently published neuropeptide prediction algorithm with a prediction probability of over 75%, and some neuropeptide proteoforms showed brain-region-specific expression (i.e., *pyya, scg2a*, and *syn1*). Gene Ontology analysis of the differentially expressed proteoforms between regions revealed region-specific biological process enrichment, i.e., innate immune response and chromatin organization in Cer, actin organization in Med, microtubule-based processes in Tele, and axonogenesis in Tec/Hab. Comparing quantitative TDP and bottom-up proteomics data from the four zebrafish brain regions revealed substantial discrepancies between proteoform-specific and protein-group-specific data sets, highlighting the value of spatially resolved TDP of brains for better understanding of protein function in a proteoform-specific manner.

## Graphical abstract

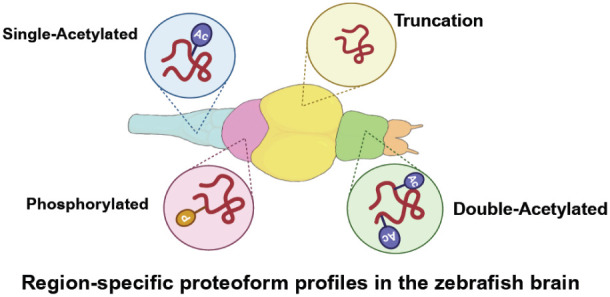

## Introduction

The human brain represents the body’s most intricate organ, consisting of approximately 86 billion neurons and an equivalent number of glial cells.^1^ This organ performs numerous specialized functions, including motor control, sensory information processing, learning, and memory consolidation. To execute these sophisticated operations, the brain displays a hierarchically organized architecture comprised of anatomically discrete yet interconnected neural regions.^2^ Substantial research efforts have focused on elucidating the molecular heterogeneity among brain regions while simultaneously characterizing their structural and functional connectivity patterns.

To achieve a more comprehensive understanding of brain function, spatially resolved proteomics is crucial, as proteins function as the main effectors of cellular mechanisms and represent the primary focus of neurological drug development. Examining the proteomic profiles of distinct brain regions, which form the core of this research, presents a promising strategy for discovering potential disease biomarkers that could advance both our understanding of region-specific brain processes and the identification of novel therapeutic targets.

Mass spectrometry (MS)-based top-down proteomics (TDP) is an optimal approach for analyzing proteoforms, which represent all forms of protein molecules from the same gene due to genetic variations, alternative splicing, and post-translational modifications (PTMs).^3^ This technique works by directly isolating and identifying intact proteoforms rather than their peptide fragments.^4–8^ Multiple research efforts have employed MS-based TDP to investigate proteoforms within brain tissue and specific brain regions.^9–15^ Research conducted by the Kelleher group identified significant variations in brain tissue proteoform patterns across four distinct healthy mouse strains.^11^ The Zhou research team found that multiple proteoforms derived from identical genes displayed varying abundance patterns when comparing hippocampal and cortical regions.^12^ The Sun lab utilized laser capture microdissection (LCM) coupled with capillary zone electrophoresis-tandem mass spectrometry (CZE-MS/MS) to compare the proteoform profiles and abundance differences between the optic tectum and telencephalon regions of the zebrafish brain, showing the brain region-specific proteoform features.^14^

MS-based TDP studies typically require high-resolution liquid-phase separations before electrospray ionization (ESI)-MS. While reversed-phase liquid chromatography (RPLC)-MS is routinely used in TDP, CZE-MS has emerged as a valuable alternative due to its high separation efficiency and sensitivity for proteoform analysis.^16–20^ In this study, we performed spatially resolved TDP of four zebrafish brain regions—medulla oblongata (Med), cerebellum (Cer), tectum/habenula (Tec/Hab), and telencephalon (Tele)—using CZE-MS/MS. We further compared these results with bottom-up proteomics (BUP) data sets of the specific brain regions.

The zebrafish (Danio rerio) serves as a powerful vertebrate model for neuroscience research due to its genetic tractability and substantial conservation with mammalian brain structure and function.^21,22^ Key brain regions, including the Tele, Cer, Tec/Hab, and Med, exhibit analogous organization between zebrafish and mammals, with approximately 70% of human genes having zebrafish orthologues. Many genes and proteins associated with neurological diseases are highly conserved, making zebrafish invaluable for studying neurodegenerative disorders and region-specific neural dysfunction.^21^ Therefore, comprehensive proteoform characterization across distinct zebrafish brain regions provides a translatable framework for understanding analogous molecular mechanisms in human brain regions, potentially revealing novel therapeutic targets and biomarkers for neurological diseases.

## Experimental Section

Adult zebrafish brains from five male AB/Tuebingen fish were collected, flash-frozen, and microdissected into four regions (Tele, Tec/Hab, Cer, and Med) under cold conditions, pooled by region, and stored at −80 °C. Proteins were extracted using urea/ABC buffer with protease and phosphatase inhibitors, homogenization, and sonication, followed by centrifugation, BCA quantification, reduction, and buffer exchange into ammonium acetate (10 mM) for top-down CZE-ESI-MS/MS analysis. Proteoforms were separated using an LPA-coated capillary on a CESI 8000 Plus system coupled to an Orbitrap Exploris 480, operating in data-dependent acquisition (DDA) mode with HCD fragmentation. RAW files were processed using the TopPIC suite^23^ for proteoform identification and label-free quantification with stringent false discovery rates (FDRs) control, and statistical analyses were performed in Perseus.^24^ Parallel quantitative BUP was carried out for the same brain regions, using CZE-ESI-MS/MS. Together, these integrated workflows enabled high-resolution, region-specific characterization of zebrafish brain proteoforms and peptides. The detailed experimental procedure is in the Supporting Information I.

## Results and Discussion

In this study, we employed CZE-MS/MS-based TDP^16,19^ to determine the proteoform profile differences across the main regions of the zebrafish brain, including Tele, combined Tec/Hab, Cer, and Med (Figure 1). Male zebrafish brains (16 months old) were used in this study. The workflow involved protein extraction through tissue homogenization and sonication in lysis buffer, followed by buffer exchange using a 10-kDa MWCO centrifugal filter device, with 250 μg of protein loaded onto each membrane. The total amount of proteoforms extracted from each brain region was about 750 μg (Tele), 1400 μg (Tec/Hab), 320 μg (Med), and 330 μg (Cer). Proteoform extracts were analyzed using dynamic pH junction-based CZE-MS/MS.^25^ To assess the reproducibility of analysis, we studied the linear correlations of proteoform intensity between replicates for each brain region (Figure S1). Scatter plots of log2-transformed intensities between replicate measurements demonstrated excellent reproducibility of CZE-MS/MS for all four zebrafish brain region samples (R^2^ = 0.89–0.97). Moreover, to assess the reproducibility of proteoform identifications, we examined the pairwise proteoform overlap between technical replicates in two brain regions (Figure S2). For Tec/Hab, the pairwise overlap ranged from 38% to 55%. For Tele, the overlap is 46–52%. The data suggest that our CZE-MS/MS system is reasonably reproducible.

**Figure 1:**
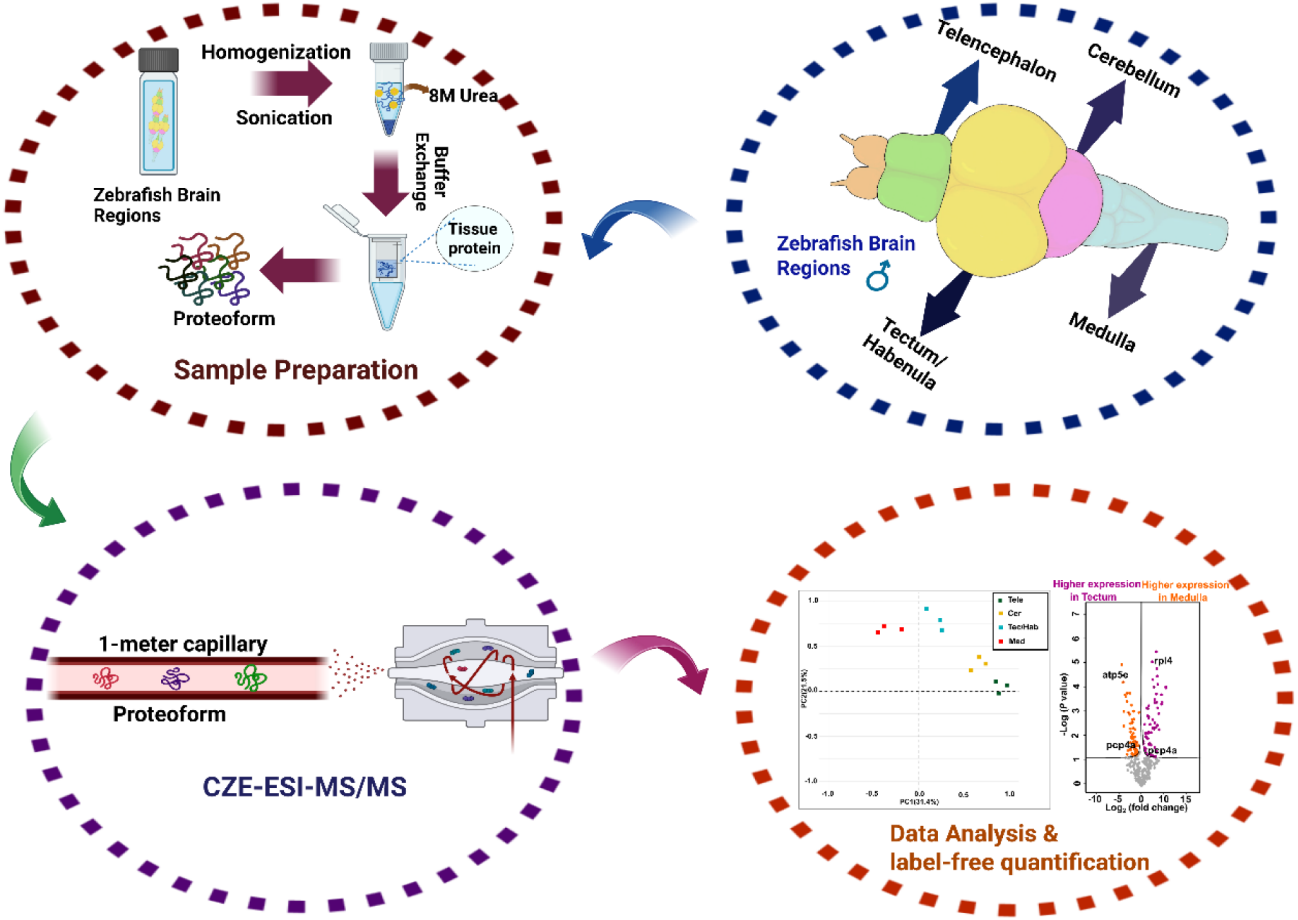
Experimental workflow for CZE-MS/MS-based top-down proteomics of zebrafish brain regions. The workflow proceeds from the **top right** and continues clockwise. **Top right**, brains from 16-month-old male zebrafish were dissected into major regions, including telencephalon (Tele), combined tectum/habenula (Tec/Hab), cerebellum (Cer), and medulla (Med). **Top left**, dissected brain tissues were homogenized and sonicated in lysis buffer to extract tissue proteins, followed by buffer exchange using a 10-kDa molecular-weight-cutoff centrifugal filter device to generate proteoform extracts. **Bottom left**, intact proteoforms were separated and analyzed using dynamic pH junction-based capillary zone electrophoresis coupled to tandem mass spectrometry (CZE-ESI-MS/MS). **Bottom right**, proteoform-level data processing, label-free quantification, and statistical analyses were performed to compare proteoform expression across brain regions. The figure is created using BioRender and is used here with permission.

### Region-Specific Proteoform Landscape in Zebrafish Brain

This study investigates region-specific proteomic changes in the zebrafish brain across four parts—Tele, combined Tec/Hab, Cer, and Med—using label-free quantitative proteomics with CZE-MS/MS. Across the four brain regions, 1,050 proteoforms were identified. Each region contains unique pools of proteoforms with only 89 proteoforms (5.1%) shared among all regions (Figure S3A). The data indicate strong region-specific proteoform expression, with only a small subset conserved across the zebrafish brain regions. In the protein-based analysis, a total of 336 proteins were identified, of which 60 proteins (17.9%) were shared across all four regions (Figure S3B). Together, these results demonstrate that while a conserved protein core is present across all zebrafish brain regions, a substantial proportion of both proteoforms and proteins are region-specific, underscoring the molecular heterogeneity of the zebrafish brain. The list of identified and quantified proteoforms is provided in Supporting Information II.

We then examined PTMs across different brain regions. We focused on four common PTMs detected in our TDP data sets, including acetylation, phosphorylation, methylation, and oxidation (Figure S4). N-terminal acetylation influences protein stability, folding dynamics, interaction capabilities, and subcellular localization patterns.^26^ Phosphorylation is a well-established regulator of cellular signaling cascades, gene expression, and neuronal communication.^27^ Methylation plays important roles in transcriptional and epigenetic regulatory networks,^28^ while oxidation can reflect redox regulation and oxidative stress–related processes. Across the four brain regions (Cer, Med, Tec/Hab, and Tele), acetylation was the most prevalent PTM, with Tele and Cer showing the highest number of acetylated proteoforms. Phosphorylated proteoforms were detected in all regions, with comparable counts but slightly elevated levels in Tele and Tec/Hab. Oxidized proteoforms were most prominent in Cer, while Med and Tele showed moderate levels. Methylated proteoforms were observed at lower abundance across all regions, with relatively consistent distribution. Overall, these results demonstrate region-specific PTM distributions in the zebrafish brain.

We analyzed proteoform intensity correlations across zebrafish brain regions. Figure 2A presents a correlation heatmap displaying Pearson correlation coefficients between technical replicates of each brain region and between brain regions: Cer, Med, Tec/Hab, and Tele. The correlation matrix reveals high reproducibility within technical replicates, with Pearson’s correlation coefficient (r) ranging from 0.81 to 1.0 for samples within the same brain region, indicating excellent experimental consistency. For inter-regional correlations, low to moderate Pearson correlation coefficients (0.3–0.8) were observed except for the Cer and Tele regions, which show strong linear correlations (r = 0.85–0.97). Figure 2B displays a principal component analysis (PCA) plot showing the separation of the four brain regions according to the differentially expressed proteoform profiles, while Figure S5 shows the PCA of the whole proteoform intensity data set. The separation among clusters reflects clearer region-specific proteoform abundance patterns in the differentially expressed subset compared to the full data set, where the regional separation is more modest. These results indicate that focusing on regulated proteoforms enhances the discriminatory power of PCA and highlights biologically meaningful variations between brain regions. Technical replicates within each region were tightly clustered in both analyses, demonstrating high reproducibility. Overall, the results illustrate distinct proteoform signatures for each brain region that align with their specialized biological functions.

**Figure 2:**
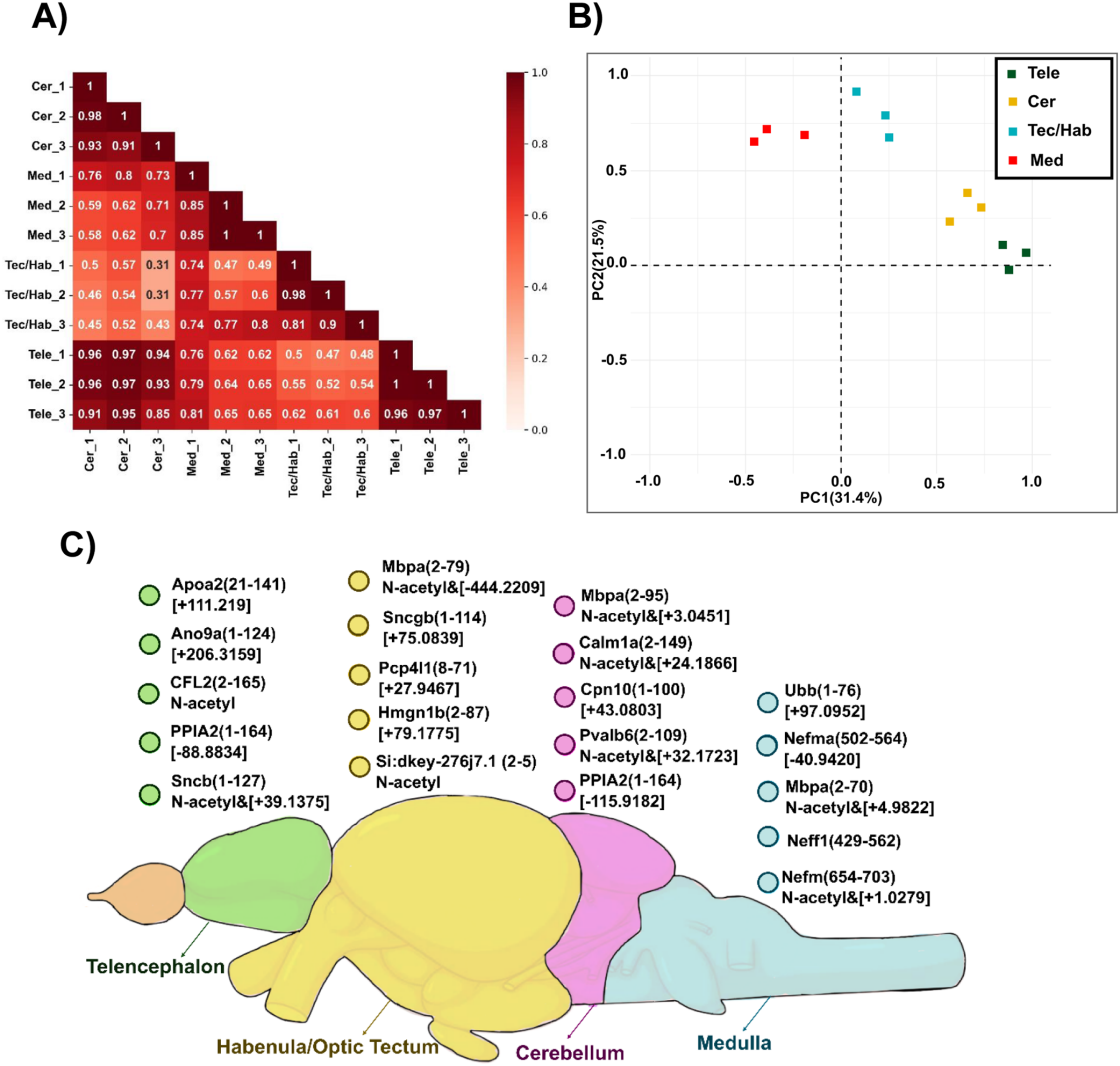
Proteoform intensity correlations and regional distribution in zebrafish brain regions. (A) Correlation heatmap showing Pearson correlation coefficients between technical replicates across four brain regions: Cer, Med, Tec/Hab, and Tele. High intraregional correlations (0.81–1.0) demonstrate excellent technical reproducibility. (B) PCA displaying separation of Tele (green), Cer (yellow), Tec/Hab (cyan), and Med (red) based on differentially expressed proteoform intensity data. PC1 and PC2 account for 31.4% and 21.5% of variance, respectively. (C) Zebrafish brain schematic showing the top five most abundant proteoforms per region with mass shifts, including apoa2 in Tele (+111.219 Da), pcp4l1 in Tec/Hab (+27.9467 Da), mbpa in Cer (N-terminal acetylation and +3.0451 Da), and ubb in Med oblongata (+97.0952 Da).

We further investigated the top five most abundant proteoforms in each region (Figure 2C). Apolipoprotein A-II (apoa2) was identified as the most abundant proteoform in the zebrafish Tele, exhibiting a +111.219 Da mass shift. The mass shift was determined and localized based on comparing the masses of observed fragment ions and the theoretical fragment ions of the unmodified protein sequence in the database. The mass shift corresponds to a modification in the region close to the C-terminus. The Tele region is responsible for sensory processing, memory, behavioral regulation, and oral regulation.^29–31^ The Tec/Hab region controls saccadic eye movements and prey-catching behaviors in zebrafish.^32,33^ In the Tec/Hab region, purkinje cell protein 4 like 1 (pcp4l1) was the most abundant proteoform, displaying a +27.0839 Da mass shift, localized primarily to the C-terminal region of the proteoform based on fragment ion evidence. The *pcp4l1* regulates calcium signaling through calmodulin interaction during brain development.^34^ Its abundance in this visually critical region suggests *pcp4l1* is essential for the rapid, calcium-mediated neural responses required for precise motor coordination and visual-guided behaviors.^35^ The Med, a critical brainstem region controlling essential survival functions,^36^ shows Polyubiquitin-B (*ubb*) as its most abundant proteoform with an N-terminal mass shift (+97.0952 Da), likely reflecting phosphorylation and oxidation modifications. The *ubb’s* prominence in this vital region underscores the critical role of the ubiquitin-proteasome system in maintaining neuronal integrity within circuits that regulate breathing, heart rate, and other involuntary functions.^37^ The *ubb* dysregulation, particularly its mutant form *ubb*+1, is strongly implicated in neurodegenerative diseases like AD.^38^ The Cer, which governs motor coordination, postural control, and motor learning in zebrafish,^39^ shows myelin basic protein (mbpa) as the most abundant proteoform with an N-terminal acetylation and an additional +3.0451 Da mass shift. The mass shift was localized close to the C-terminus of the proteoform sequence. *Mbpa’s* dominance reflects the cerebellum’s dense myelinated networks required for rapid signal transmission and complex sensory integration. As an essential myelin sheath component, *mbpa* enables the sophisticated neural circuitry underlying cerebellar functions from basic motor control to advanced behavioral adaptations, social behaviors, and emotional responses, emphasizing the critical role of proper myelination in cerebellar-mediated learning and coordination.^40^

### Quantitative TDP of Zebrafish Brains Revealed Substantial Proteoform Abundance Differences across Brain Regions

We performed quantitative TDP analysis across the four zebrafish brain regions (Cer, Med, Tec/Hab, and Tele) using label-free quantification to identify differentially expressed proteoforms. Comparative proteoform quantification revealed region-specific expression patterns. For Cer comparisons, we quantified 323, 330, and 285 proteoforms from 140, 157, and 131 genes when compared to Med, Tele, and Tec/Hab, respectively. Among these, 67, 70, and 44 proteoforms showed statistically significant differences in abundance (Figures 3A, S6A, and B **).** When we compared Med with Tec/Hab and Tele, 120 and 230 differentially expressed proteoforms were determined, respectively (Figure S6C and D **).** The Tec/Hab versus Tele comparison identified 359 proteoforms from 160 genes, with 175 proteoforms exhibiting significant abundance differences (Figure 3B). The lists of differentially expressed proteoforms are shown in Supporting Information II.

**Figure 3:**
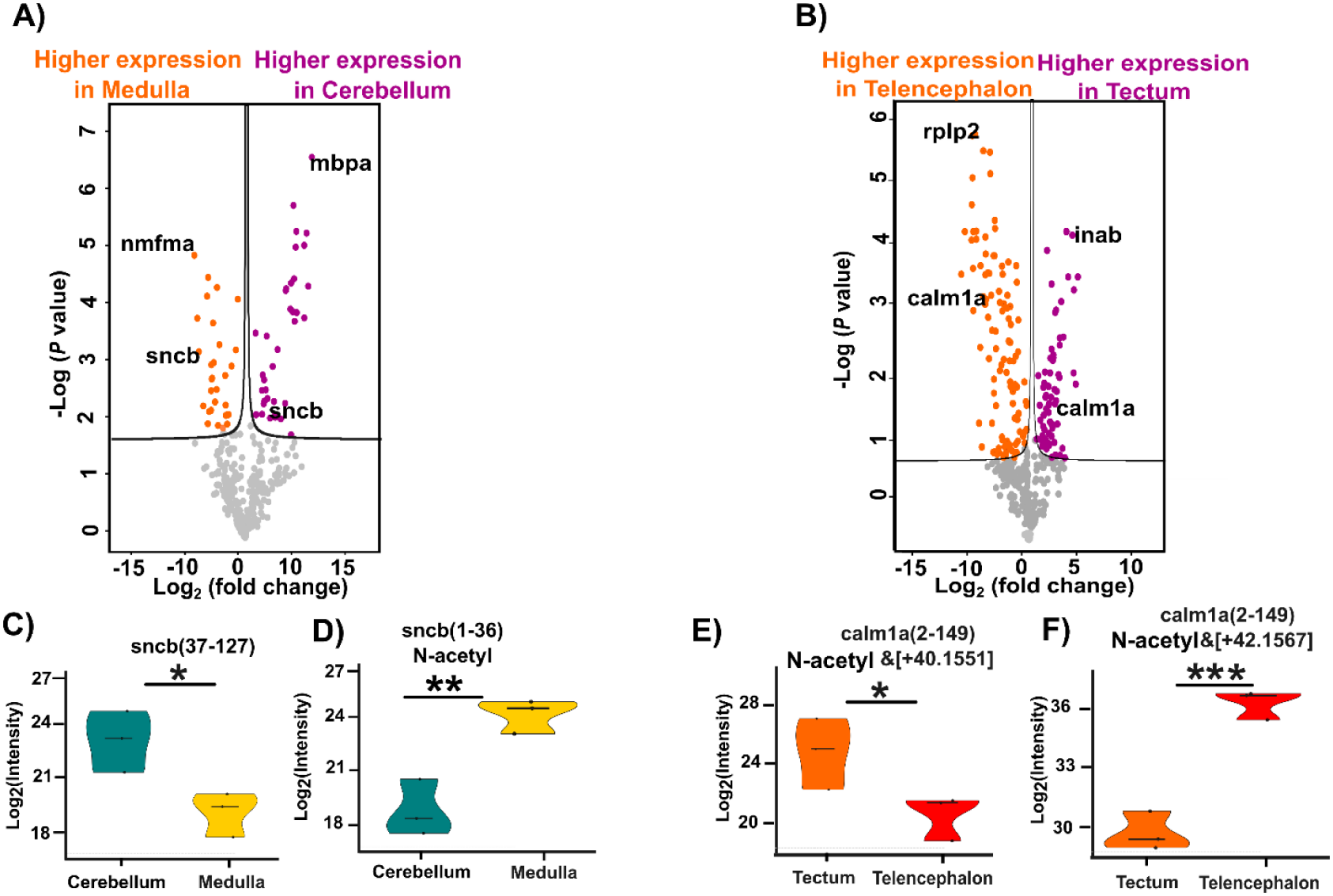
Summary of label-free quantification (LFQ) data comparing brain regions. (A) Volcano plot visualization of quantified proteoforms (323) showing higher-abundance proteoforms in Med (orange) and Cer (purple). (B) Volcano plot visualization of quantified proteoforms (359) showing higher abundance proteoforms in Tele (orange) and Tec/Hab (purple). Selected differential proteoforms are labeled with their corresponding gene names. Panels (A) and (B) were generated using Perseus software with parameters set to S0 = 0.1 and FDR = 0.1. Violin plots illustrate abundance differences of (C) sncb (37–127) proteoform between Cer and Med, (D) sncb (1–36) N-acetyl proteoform between Cer and Med, (E) calm1a (2–149) N-acetyl proteoform between Tec/Hab and Tele, and (F) calm1a (2–149) N-acetyl proteoform between Tec/Hab and Tele. Statistical significance: * *p* < 0.05, ** *p* < 0.01, *** *p* < 0.001.

Interestingly, we found that proteoforms from the same genes (i.e., *sncb*, *calm1a*, *mbpa, pcp4l1, apoa2*, and *nefma*) can have opposite expression patterns between two brain regions. For example, when comparing Tele and Tec/Hab, four *calm1a* proteoforms exhibited higher expression in Tele, while one showed increased expression in Tec/Hab. Similarly, when comparing Cer and Tec/Hab, three *mbpa* proteoforms were more abundant in Cer, while one was elevated in Tec/Hab, highlighting the value of TDP for proteoform-specific measurements.

We next investigated several differentially expressed proteoforms of six critical proteins—Synuclein Beta (*sncb*), Calmodulin (*calm1a*), Myelin Basic Protein A (*mbpa*), Purkinje Cell Protein 4 Like 1 (*pcp4l1*), Apolipoprotein A2 (*apoa2*), and Neurofilament Medium Chain A (*nefma*)—across different brain regions. Synucleins have been extensively studied for their roles in neurodegenerative diseases such as Parkinson’s disease (PD) and AD.^41,42^ Both AD and late-stage PD patients show deficits in cognitive functions, including memory. Interestingly, we identified two β-synuclein proteoforms: a truncated form that was more abundant in the Cer compared to Med [*log*_2_ (*proteoform intensity fold change*) (*FD*) *=* *4.69, p-value = 0.015*] and an N-terminally acetylated form that was higher in the Med (*FD* *= −5.9, p-value = 0.002*), Figure 3C–D. Across all four brain regions, the N-terminally acetylated β-synuclein proteoform was most abundant in Med, and had the lowest abundance in Cer. In contrast, the truncated β-synuclein proteoform had a comparable abundance in Tec/Hab, Tele, and Cer, but substantially lower in Med. The data further supports the opposing regional distribution patterns of these two proteoforms.

The Cer plays a central role in motor coordination and synaptic plasticity, while the Med is involved in autonomic control and signal transmission between the brain and spinal cord. These findings suggest that distinct β-synuclein proteoforms may contribute to region-specific neuronal functions. Calmodulin is a critical calcium-sensing protein that regulates numerous cellular processes, and its dysregulation is implicated in the amyloid pathway and tangle formation in AD.^43^ Our data revealed a striking example of bidirectional regulation for this protein. As evidenced in Figure 3E–F, we identified two distinct *Calm1a* proteoforms with opposite expression patterns between Tec/Hab and Tele (*FD = 4.5, p-value =* 0.032 *and FD = −5.07, p-value = 0.00068*). This bidirectional regulation suggests that different Calm1a proteoforms may play distinct roles in calcium signaling pathways depending on the neuronal context of specific brain regions. Across all four brain regions, the N-terminally acetylated Calm1a proteoform (+42.1567 Da) was most abundant in Tele, followed by Cer and Med, with the lowest level in Tec/Hab. In contrast, the second Calm1a proteoform (+40.1551 Da) had substantially higher abundance in Tec/Hab than other three brain regions. Similar bidirectional regulation was also observed for proteoforms of *pcp4l1, mbpa, nefma*, and *apoa2*, Figure S7A–H. Neurofilament light chain (*nefl*) is crucial for the structure of the neuronal cytoskeleton, forming part of neurofilaments alongside the neurofilament medium chain (*nefm*) and neurofilament heavy chain (*nefh*). *Nefl* is essential for neurofilament assembly, which regulates axonal caliber outgrowth, regeneration, guidance, and nerve impulse conduction. In AD, abnormal *nefl* expression disrupts the neuronal cytoskeleton, leading to cognitive decline, and memory loss.^44,45^ Overall, the observed region-specific expression patterns indicate that distinct proteoforms of these proteins may perform specialized molecular functions within different brain regions. Such spatial proteoform diversity highlights the importance of proteoform-resolved analysis for understanding localized neuronal processes and functional specialization within the brain.

We then assessed neuropeptide-like proteoforms across the four zebrafish brain regions using a machine-learning-based neuropeptide prediction model,^46^ which has demonstrated strong performance and yielded 153 neuropeptide candidates with a prediction probability of over 75%. Complete proteoform data are provided in Supporting Information II. Neuropeptides are highly diverse signaling molecules that play critical physiological roles and can serve as biomarkers for disease states, including AD, cancers, and environmental stress.^47,48^ As chemical indicators of internal states, they modulate nearly all physiological functions, including appetite, blood glucose levels, blood pressure, inflammation, and anxiety.^49^ Their diverse nature—ranging from 3 to 70 amino acids with complex PTMs and isoform variations—makes them valuable yet challenging targets for biomarker discovery and therapeutic applications.^50,51^ Analysis of physicochemical properties revealed key characteristics of the identified neuropeptide candidates. The isoelectric points (pI) distribution showed that most neuropeptides fell within an acidic to neutral range (pI ∼ 4–6), consistent with the charged, soluble nature expected for secreted signaling peptides Figure S8A. The grand average of hydropathicity (GRAVY) scores indicated a general tendency toward hydrophilicity (Figure S8B), a favorable trait for drug development due to improved solubility. The mass distribution reveals that the majority of precursors cluster between ∼2.5 and 10 kDa, matching the typical size range of neuropeptides. Together, these profiles are consistent with previously reported neuropeptide characteristics and support the conclusion that the identified proteoforms exhibit features expected of true neuropeptide candidates.^52–54^ In our neuropeptide-like proteoforms, many of them were produced by cleavages at the basic residues—Arg, Lys, and His. This pattern closely aligns with previously reported prohormone-processing motifs, supporting the conserved basic-residue–driven cleavage mechanisms described in neuropeptide precursor research.^55^

Among the predicted neuropeptide-like proteoforms, we selected *pyya*, *scg2a*, and *syn1* for detailed analysis, focusing specifically on the proteoforms detected for each (Figure S9A–C). Peptide YY (*pyy*) functions as a satiety hormone released from the gut after feeding, signaling fullness to the brain by acting on neuropeptide Y receptors—particularly the Y2 receptor.^56,57^ Secretogranin-2a (*scg2a*) serves as a precursor for the neuropeptide secretoneurin (SN), which regulates nervous, endocrine, and immune functions and contributes to neuroprotection and neuroinflammation. SN is produced through proteolytic cleavage of *scg2* and acts by binding specific receptors, enabling *scg2* to influence processes such as neurotransmitter release, immune cell migration, and mating behavior via estrogen-related signaling.^58,59^ An N-terminally acetylated proteoform of synapsin-1 isoform X1 (syn1) showed a +80-Da mass shift, consistent with a single phosphorylation event likely occurring between Ser11 and Leu17, most probably at Ser11 or Ser15—a modification corresponding to the canonical site 1 phosphorylation in domain A.^60,61^ Phosphorylation at this site, mediated by PKA and CaMKI/IV, is known to promote dissociation of synapsin I from synaptic vesicles, facilitating vesicle mobilization from the reserve pool to the readily releasable pool and modulating activity-dependent neurotransmitter release.^62^ Synapsin I thereby plays a central role in synaptic function and plasticity through its dynamic interaction with synaptic vesicles.^63^ Overall, this integrative analysis reveals a rich landscape of neuropeptide-like proteoforms in the zebrafish brain, supporting their functional significance and value as promising biomarker candidates.

To assess the broader biological context of region-specific proteoform alterations, we performed Gene Ontology (GO) enrichment analysis using the DAVID platform.^64^ GO analysis is gene-centric and does not reveal functional differences among individual proteoforms derived from the same gene. Therefore, the enrichment results should be interpreted as reflecting gene-level biological associations rather than definitive functional assignments of specific proteoforms. Across brain regions, enriched biological processes were generally consistent with known regional functions. For each brain region, we obtained a list of proteoforms by combining the differentially expressed proteoforms from comparisons with the other three brain regions. For example, for Cer, we combined the lists of differentially expressed proteoforms in Figures 3A, S6A, and B. The genes corresponding to these differentially expressed proteoforms for each specific brain region were used for the GO enrichment analysis. For the Cer, the top enriched processes included disruption of plasma membrane integrity in another organism, defense response to Gram-positive bacteria, heterochromatin formation, and innate immune response (Figure S10A). Notably, two distinct histone H2A proteoforms were detected: one N-terminally acetylated and another with a +83.0845-Da mass shift. H2A variants such as *H2A.Z* serve as epigenetic regulators whose dysregulation links to neurological disorders, including AD, PD, and cerebellar ataxias.^65,66^ In the Cer, *H2A.Z* regulates mitochondrial gene expression, with its absence causing progressive ataxia and neurodegeneration.^66^ For the Tec/Hab brain regions, the enriched biological process (BP) categories include regulation of microtubule depolymerization, clustering of voltage-gated sodium channels, axonogenesis, and actin filament organization (Figure S10B). Thymosin beta (*Tβ*) protein regulates actin filament organization and exhibits seven distinct proteoforms with differential expression in the Tec/Hab region. Five proteoforms display N-terminal acetylation with varying mass shifts, including one with a +80.0153 Da modification that may arise from phosphorylation. Additionally, one full-length proteoform shows a −218.0251-Da mass shift. *Tβ* regulates neuronal growth and differentiation^67,68^ and controls optic tectum development through actin cytoskeleton regulation, driving neural stem cell proliferation and neurogenesis.^69^ GO analysis of Tele regions showed significant enrichment in microtubule-based processes (Figure S10C). This enrichment encompassed two N-terminally acetylated proteoforms and one complete proteoform with a +91.1639 Da mass shift from dynein light chain, along with one truncated tubulin alpha chain proteoform. Dynein light chains are essential for telencephalic development, regulating neurogenesis, nuclear migration, and neuronal positioning.^70–72^ Med analysis demonstrated significant enrichment in axonogenesis as the predominant process in this region. This enrichment comprised five truncated proteoforms of Microtubule-associated protein 1A isoform X2 and one truncated proteoform of Dihydropyrimidinase-related protein 2 isoform X1 (*drp-2*) (Figure S10D). Dihydropyrimidinase-related protein 2 (*dpysl2/crmp2*) isoform X1 is upregulated in the Med following nerve injury, where it facilitates neuronal repair through cytoskeletal remodeling and microtubule assembly. The protein promotes axonal regrowth by binding tubulin dimers while regulating growth cone dynamics and synaptic plasticity through ion channel trafficking, making it essential for the medulla’s neural repair and regeneration capacity.^73,74^ Collectively, these findings suggest that genes corresponding to region-specific proteoform alterations are associated with biological processes consistent with established regional functions. However, further studies are required to elucidate the functional contributions of individual proteoforms.

### Comparing BUP and TDP Data of Zebrafish Brain Regions

We also carried out quantitative BUP of zebrafish brain regions (Tele, Tec/Hab, Cer, and Med). Our objectives were 2-fold: (i) to obtain a comprehensive overview of region-specific protein group expression, and (ii) to compare and integrate BUP and TDP quantification results to gain deeper insight into brain region–specific proteomic regulation. To characterize the regional specificity and commonality of protein expression across brain regions, we analyzed the overlap of proteins and peptides identified in the Cer, Med, Tec/Hab, and Tele (Figure S11A and B **).** Protein overlap analysis revealed that 335 proteins (27% of the total) were expressed in all four brain regions, suggesting a core set of proteins essential for fundamental neuronal functions. The remaining proteins showed varying degrees of regional specificity, with the Tele exhibiting the smallest region-specific protein population (38 proteins, 3%), while other regions displayed moderate specificity, ranging from 52 to 119 proteins. Correlation analysis of protein group intensities between technical replicates showed excellent reproducibility (correlation coefficients >0.8) and revealed distinct expression patterns between brain regions (Figure S11C). This regional distinctiveness was further confirmed by t-SNE analysis, which demonstrated clear clustering of technical replicates by anatomical region (Figure S11D), indicating that each brain area possesses a characteristic proteomic signature that distinguishes it from other regions.

In our experiment, we quantified 657, 851, and 881 protein groups in the Cer when compared to the Med, Tele, and Tec/Hab, respectively (Figures 4A, S12A, and B **).** Volcano plots were generated using *t* test cutoff values of FDR 0.05 and S0 0.1. Complete differential expression data are provided in Supporting Information II. We identified 21, 28, and 10 protein groups upregulated in the Cer, whereas 548, 769, and 844 protein groups were upregulated in the Med, Tele, and Tec/Hab, respectively. In comparisons involving the Med, 950 and 942 protein groups were quantified against the Tec/Hab and Tele, with 785 and 656 showing significant differences, respectively (Figure S12, C–D). Finally, the comparison between the Tec/Hab and Tele revealed 1,017 protein groups, of which 700 exhibited significant differential expression (Figure 4B). GO enrichment analysis of highly expressed proteins revealed distinct biological processes across brain regions. In the Cer, enriched pathways were mainly associated with central nervous system myelination and tight junction organization, consistent with our previous top-down findings. Key proteins involved in these processes include *dmalpha2b*, *dmgamma1*, and multiple claudins, which contribute to blood–brain barrier integrity and myelin structure.^75,76^ In the Med, enriched terms reflected functions linked to sensory axon fasciculation and receptor protein-tyrosine kinase signaling, processes essential for autonomic neuron activity and medullary circuit development.^77–79^ The Tec/Hab showed enrichment of pathways related to cytoskeletal and filament organization, including actin and intermediate filament dynamics. These findings align with our previous TDP data, with vimentin exemplifying proteins involved in structural organization and injury response.^80–82^ In the Tele, enriched GO categories included neuron projection development, axon development, translation, and synapse organization, reflecting active neural growth and maturation. Proteins such as adcyap1b play key roles in these developmental and synaptic.^83–85^

**Figure 4:**
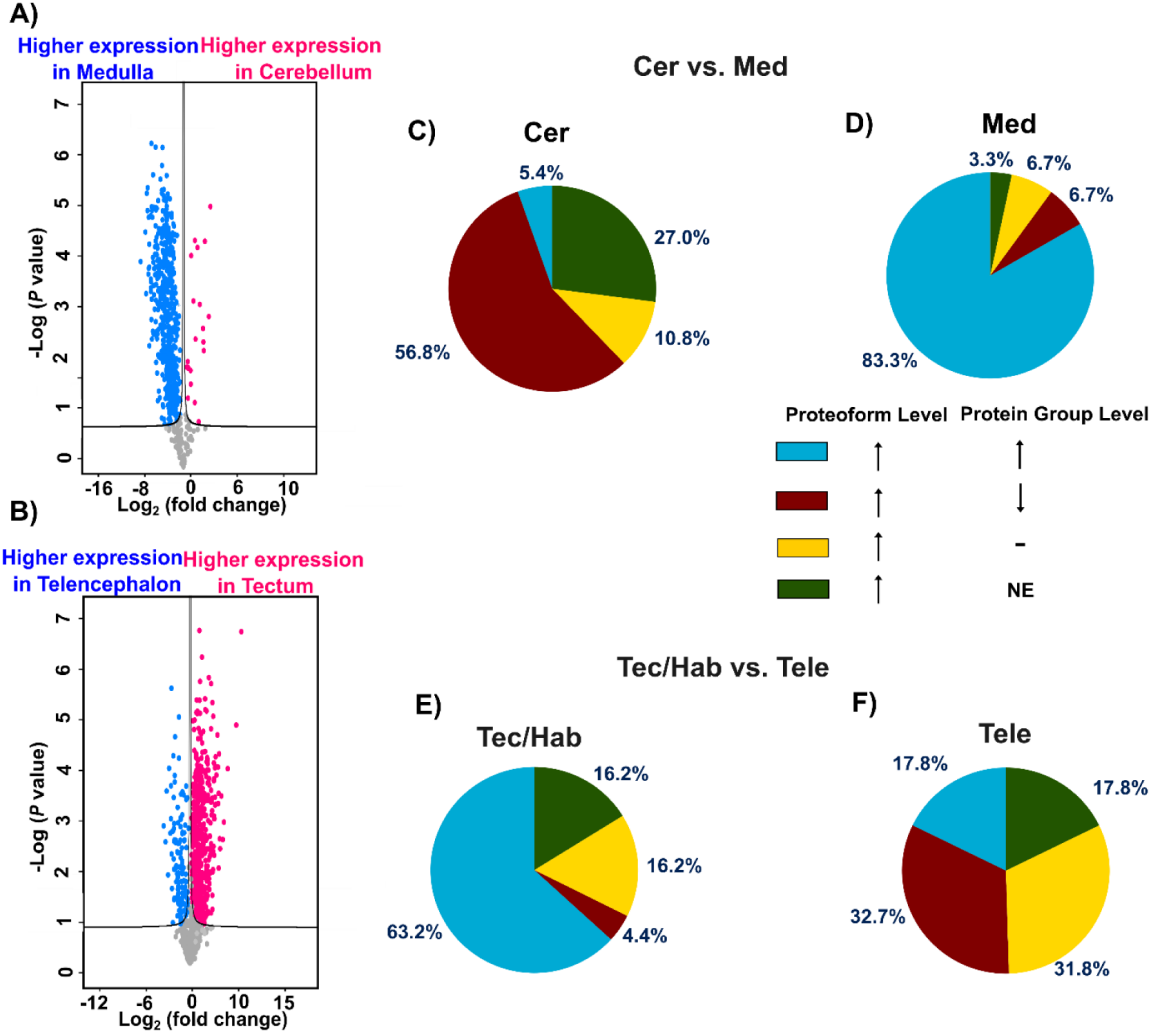
Comparative analysis of quantitative BUP and TDP data to provide a comprehensive assessment of gene expression products at both protein group and proteoform levels. Volcano plots display protein group-level quantification from BUP analysis comparing (A) Med versus Cer and (B) Tele versus Tec/Hab regions in zebrafish brains, with statistical significance determined using *t* test parameters of false discovery rate (FDR) = 0.05 and S0 = 0.1. In panel A, blue dots indicate protein groups highly expressed in the Med, while red dots indicate protein groups with higher abundance in the Cer. In panel B, blue dots indicate protein groups highly expressed in the Tele, whereas red dots indicate protein groups with higher abundance in the Tec/Hab. Cross-platform comparison of quantitative results between TDP-derived proteoform-level measurements and BUP-derived protein group–level measurements for differentially expressed targets across zebrafish brain regions: (C) Cer, (D) Med, (E) Tec/Hab, and (F) Tele. Panels C and D show proteoforms differentially expressed in the Cer vs Med comparison, highlighting those with higher abundance in Cer and Med, respectively. Panels E and F show proteoforms differentially expressed in the Tec/Hab vs Tele comparison, highlighting those highly expressed in Tec/Hab and Tele, respectively. Color coding represents expression patterns at proteoform vs protein group levels. “NE” indicates proteins that did not exist in the respective analysis; “-” denotes proteins showing no statistically significant difference in expression.

To evaluate the concordance between TDP and BUP quantification approaches, protein accession numbers from differentially expressed proteoforms identified by TDP were cross-referenced with corresponding protein groups quantified by BUP across distinct zebrafish brain regions. The analysis revealed substantial discrepancies between BUP and TDP across all regional comparisons. Figure 4C and D highlight proteoforms with higher expression in the Cer and Med, respectively. For 10.8% and 6.7% of proteoforms exhibiting significantly elevated abundance in Cer and Med, respectively, the BUP data showed no significant expression difference at the protein group level (Figure 4C and D). For 27% and 3.3% of proteoforms with higher abundance in Cer and Med, respectively, the corresponding proteins were not identified by BUP analysis of the specific brain region. For example, multiple proteoforms of β-synuclein, Parvalbumin-7, and Histone H2A, each bearing distinct PTMs, demonstrated statistically significant abundance differences in TDP analysis. However, these proteins were not identified by BUP. Notably, the 27% in Cer and 3.3% in Med were significantly reduced to 16% and 0% when compared to the combined BUP protein group data of all brain regions. Concordance between TDP and BUP data was observed for 83.3% and 5.4% of highly expressed proteoforms in Med and Cer, respectively, while opposing expression patterns were detected for 56.8% and 6.7% of proteoforms. Similarly, we also observed obvious discrepancies between BUP and TDP in the comparisons of Tele vs Tec/Hab (Figure 4E and F) and in other comparisons (Figure S13). For example, more than 20% and 60% of proteoforms with higher expression in Tec/Hab and Tele show disagreement between BUP and TDP data (Figure 4E and F). Additionally, proteoforms of synaptosomal-associated protein 25-B, thymosin beta, and vesicle-associated membrane protein 3 with various PTMs showed significant abundance differences in TDP data between Tec/Hab and Tele. However, the corresponding proteins were not identified by BUP of the specific brain regions.

These discrepancies reflect fundamental differences in analytical approaches, where BUP quantification represents the average abundance of all proteoforms derived from a single gene, losing proteoform-specific information through enzymatic digestion, whereas TDP directly characterizes individual proteoforms. Given that proteins undergo substantial PTM changes independent of total protein abundance alterations across biological processes, differential proteoform abundance detected by TDP may occur without corresponding changes in overall protein levels measured by BUP. This comparative analysis demonstrates two critical insights: the complementary nature of TDP and BUP approaches provides comprehensive coverage of gene expression products across spatially distinct zebrafish brain regions. At the same time, the observed discrepancies underscore the need for proteoform-specific protein characterization using TDP to interpret protein roles in biological processes accurately.

## Conclusions

In this work, we established a spatially resolved TDP workflow using CZE-MS/MS to characterize proteoform diversity across four anatomically distinct zebrafish brain regions. Our analysis identified 1,050 proteoforms and revealed pronounced region-specific proteoform expression, with only a small fraction shared across all regions. These proteoform-level differences were strongly associated with the specialized biological functions of each brain region, as demonstrated by enriched pathways related to myelination and chromatin regulation in the Cer, cytoskeletal remodeling in the Tec/Hab, microtubule-based processes in the Tele, and axonogenesis in the Med. We further uncovered extensive bidirectional regulation of proteoforms originating from the same genes—such as *sncb, calm1a, mbpa, pcp4l1, apoa2*, and *nefma*—highlighting the importance of proteoform-specific measurements when studying brain molecular heterogeneity. Our analysis identified nearly 200 neuropeptide-like proteoforms, including region-specific forms of *pyya, scg2a*, and *syn1*, underscoring TDP’s ability to reveal heterogeneity in neuropeptide-like proteoforms that may underline region-specific signaling functions. Finally, the cross-platform comparison revealed substantial discrepancies between the TDP and BUP data sets, underscoring that protein-group–level quantification often masks underlying proteoform changes driven by PTMs and sequence variations. Together, these findings underscore the unique value of TDP for resolving the molecular complexity of the brain and provide a foundational proteoform atlas that can facilitate future studies of neural function, plasticity, and neurodegenerative disease mechanisms.

Despite the strengths of this study, there are a few limitations. First, we identified only a limited number of proteoforms, suggesting we captured only part of the true complexity in the zebrafish brain. Second, our spatial resolution was limited because we analyzed large brain regions rather than smaller areas or individual cell types, so some fine-scale differences may have been missed. In the future, improvements in instrument sensitivity, separation and fragmentation methods, and higher-resolution sampling—such as using laser-capture microdissection—could help identify more proteoforms and provide more detailed spatial information. Combining spatial TDP with techniques like single-cell proteomics or PTM-focused enrichment will also help us better understand proteoform changes in the brain.

## Supplementary Material





## Data Availability

The MS raw data have been deposited to the ProteomeXchange Consortium via the PRIDE partner^86^ repository with the data set identifier PXD070925.

## Notes

Supplemental experimental section; mass, estimated volume, and lysis buffer volume of dissected zebrafish brain regions (Table S1); reproducibility of proteoform intensity measurements between technical replicates of CZE-MS/MS across four zebrafish brain regions (Figure S1); proteoform overlap between biological replicates (Figure S2); upSet plots summarize the distribution of proteoforms and proteins across zebrafish brain regions (Figure S3); distributions of proteoforms carrying common PTMs (Figure S4); principal component analysis of proteoform intensity profiles across zebrafish brain regions (Figure S5); volcano plot analysis of differentially expressed proteoforms across zebrafish brain regions (Figure S6); violin plots showing bidirectional proteoform regulation across zebrafish brain regions (Figure S7); distribution of physicochemical properties of predicted neuropeptides (Figure S8); amino acid sequences and MS/MS fragmentation patterns of three identified proteoforms (Figure S9); Gene Ontology (GO) enrichment analysis of genes corresponding to differentially expressed proteoforms (Figure S10); summary of protein groups and peptides from BUP analysis of different brain regions (Figure S11); volcano plots showing the differentially expressed protein groups from quantitative BUP (Figure S12); comparative analysis of quantitative BUP and TDP data for comprehensive assessment of gene expression products at protein group and proteoform levels (Figure S13) (PDF)
Lists of identified and quantified proteoforms and protein groups from TDP and BUP analysis of the zebrafish brain regions (XLSX)
