# Supplementary material for: Top-Down Proteomics of Zebrafish Brain Regions Using Capillary Zone Electrophoresis-Tandem Mass Spectrometry
Source: J Proteome Res. 2026 Apr 17;25(5):2546–57. doi: 10.1021/acs.jproteome.6c00007 (PMC13140148; doi:10.1021/acs.jproteome.6c00007)
Supplement: Supplementary file 1 [file pr6c00007_si_001.pdf]

Top-Down Proteomics of Zebrafish Brain Regions Using Capillary Zone Electrophoresis-Tandem Mass Spectrometry

Mehrdad Falamarzi Askarani,[1] William Poulos[2], Maryam Rahimzadeh Dashtaki,[1] Fei Fang[1], Jose B. Cibelli,[2,3], Liangliang Sun[1, *]

[1]Department of Chemistry, Michigan State University, 578 S Shaw Lane, East Lansing, Michigan 48824, USA

[2]Department of Animal Science, Michigan State University, East Lansing, MI 48824, USA.

[3]Department of Large Animal Clinical Sciences, Michigan State University, East Lansing, MI 48824, USA.

* Corresponding Authors.

L. Sun, Email: lsun@chemistry.msu.edu; Phone: 517-353-0498

TABLE OF CONTENTS

Section	Page
**Supplemental Experimental Section**	**S3-S7**
Materials and Reagents	**S3**
Tissue Preparation	**S3**
Sample Preparation for CZE-MS/MS	**S4**
CZE-MS/MS Analysis	**S4**
Database Search for Proteoform Identification and Quantification	**S5**
Quantitative Bottom-Up Proteomics (BUP) of Zebrafish Brain Regions	**S6**
**Table S1.** Summary of mass, volume, and lysis buffer volume of dissected Zebrafish Brain Regions	**S8**
**Figure S1.** Reproducibility Assessment	**S9**
**Figure S2**. Proteoform Overlap	**S10**
**Figure S3.** UpSet Plots	**S11**
**Figure S4**. PTM Distribution	**S12**
**Figure S5.** PCA Analysis	**S13**
**Figure S6.** Volcano Plots	**S14**
**Figure S7.** Violin Plots	**S15**
**Figure S8**. Physicochemical Properties	**S16**
**Figure S9.** MS/MS Fragmentation Patterns	**S17**
**Figure S10.** GO Enrichment Analysis	**S18**
**Figure S11.** Protein groups and Peptide Overlaps	**S19**
**Figure S12.** BUP Volcano Plots	**S20**
**Figure S13.** BUP vs TDP Comparisons	**S21**
**References**	**S22-S23**
**Proteoforms/protein from TDP & BUP analysis of zebrafish brain regions**	**Separate (XLSX)**

Experimental Section

Materials and Reagents

Ammonium bicarbonate (ABC), dithiothreitol (DTT), and Amicon Ultra centrifugal filter units (0.5 mL, 10 kDa molecular weight cut-off) were obtained from Sigma-Aldrich (St. Louis, MO). Fisher Scientific (Pittsburgh, PA) supplied LC/MS grade water, LC-grade acetic acid (AA), methanol (MeOH), and bare fused silica capillaries (50 μm inner diameter, 360 μm outer diameter, Polymicro Technologies). Acrylamide was procured from Acros Organics (Fair Lawn, NJ). Complete mini protease inhibitor cocktail and PhosSTOP (EASYpacks) were acquired from Roche (Indianapolis, IN).

Tissue preparation

Adult zebrafish brains were collected from five mature male zebrafish (AB/Tuebingen line). The zebrafish were provided by Professor Jose Cibelli's group at the Department of Animal Science at Michigan State University. All animal-related experiments were performed using a protocol approved by the Institutional Animal Care and Use Committee of Michigan State University.

Following euthanasia with tricaine overdose (0.4%), whole brains were carefully extracted using standard microdissection techniques and placed individually on small aluminum foil squares (approximately 1.5 cm^2). Each brain was immediately flash-frozen by immersion in liquid nitrogen for approximately 30 seconds to preserve tissue integrity and structural morphology during subsequent regional dissection. The rapid freezing protocol was employed to minimize protein degradation and maintain the architectural integrity of distinct brain regions, which is necessary for accurate anatomical separation.

The frozen brains were then transferred to a pre-chilled dissection surface consisting of 100 mm petri dishes filled with water and frozen to maintain a consistent low temperature during manipulation. Using pre-chilled microdissection tools (forceps, scalpels, and micro-scissors maintained on ice) under an illuminated dissecting microscope, each frozen brain was manually dissected into four distinct neuroanatomical regions based on established zebrafish brain atlas landmarks: telencephalon (Tele), combined habenula-optic tectum (Tec/Hab), cerebellum (Cer), and medulla (Med). The olfactory bulbs were excluded from analysis due to insufficient tissue volume for downstream proteomic analysis. The habenula and optic tectum were combined as a single sample due to the technical challenges associated with accurately separating these closely associated midline structures without compromising sample integrity.

Dissected regions of the same anatomical type were pooled from all five fish into individual pre-labeled 5 mL round-bottom tubes maintained on ice throughout the procedure. All pooled samples were immediately stored at -80°C until protein extraction and subsequent analysis by CZE-MS/MS analysis. This pooling strategy ensured sufficient protein yield for comprehensive proteomics characterization.

Sample Preparation for CZE-MS/MS

For protein extraction, adult zebrafish brain regions (Tele, combined Tec/Hab, Cer, and Med) were lysed in a solution containing 8 M urea and 100 mM ABC at pH 8.0, with the addition of complete protease inhibitor mixture and PhosSTOP phosphatase inhibitors. The volume of the lysis buffer shown in **Table S1** was determined according to the mass of the brain region to maintain the ratio between lysis buffer volume and mass of brain region around 20 (i.e., ~20 µL lysis buffer per 1 mg tissue). Brain tissue homogenization was performed (~2 min) using a Fisher Scientific Homogenizer 150, followed by ice sonication (~10 min) with a VWR Scientific Branson Sonifier 250 to enhance protein extraction. Following centrifugation at 16,000 × g for 10 minutes at 15°C, lipids and cellular debris were removed, and the protein-rich supernatant was recovered. Protein quantification was accomplished using a bicinchoninic acid (BCA) assay from Fisher Scientific (Pittsburgh, PA). The protein concentration was 2.07 mg/mL (Tele), 1.4 mg/mL (Tec/Hab), 1.28 mg/mL (Med), and 0.76 mg/mL (Cer). The total amount of proteins extracted from each brain region was about 750 µg (Tele), 1400 µg (Tec/Hab), 320 µg (Med), and 330 µg (Cer). Before CZE-MS/MS analysis, zebrafish brain proteins underwent reduction with 5 mM DTT for 30 minutes at 37°C. Buffer exchange and urea removal were performed using an Amicon Ultra Centrifugal Filter with a 10 kDa molecular weight cutoff (MWCO) with 250 µg of protein loaded onto each membrane. The filter was first conditioned by adding 200 µL of 10 mM ammonium acetate (pH 6.9) and centrifuging at 14,000 × g for 15 min. The sample was then loaded onto a Microcon-10 kDa centrifugal filter (Sigma-Aldrich) and centrifuged at 14,000 × g for 20 min to facilitate urea removal. To ensure complete buffer exchange, the retained sample was subjected to three sequential washes with 10 mM ammonium acetate (pH 6.9), each followed by centrifugation under the same conditions. The retained proteoform sample was reconstituted in 30-40 µL of 10 mM ammonium acetate (pH 6.9) for downstream analysis. For each brain region, tissue from five male brains was pooled and processed in three independent buffer exchange aliquots; the resulting samples were then reunited and analyzed by CZE/MS/MS in technical triplicate. Final protein concentration after buffer exchange was determined using a BCA kit according to the manufacturer's instructions: ~ 3.0 mg/mL (Tele), 5.2 mg/mL (Tec/Hab), 4.0 mg/mL (Med), and 4.0 mg/mL (Cer). The proteoform samples were subjected to CZE-MS/MS analysis in technical triplicate, with approximately 200 ng loaded per run.

CZE-MS/MS Analysis

CZE-MS/MS was conducted using a Beckman Coulter CESI 8000 Plus CE system coupled to a Thermo Fisher Scientific Orbitrap Exploris 480 mass spectrometer, utilizing a custom-built electrokinetically driven sheath-flow CE-MS nanospray interface[1,2]. A 1-meter-long fused silica capillary (50 μm inner diameter, 360 μm outer diameter) was coated with linear polyacrylamide (LPA) following previously established protocols[3] . One end of the capillary was tapered to an outer diameter of 70–80 μm using hydrofluoric acid etching[4]. Sample injection was performed by applying a 5-psi pressure for 17 seconds, delivering approximately 100 nL of sample, consistent with Poiseuille's law. Electrophoretic separation was achieved by applying a 20 kV voltage at the injection end for 70 minutes, while a 2 kV potential was applied at the CE-MS interface to initiate ESI. Between runs, the capillary was flushed for 10 minutes at 50 psi with the background electrolyte (BGE). The ESI emitter was fabricated from a glass capillary (0.75 mm i.d., 1.0 mm o.d., 10 cm length) using a Sutter P-1000 micropipette puller, resulting in an orifice diameter of 20–40 μm. The BGE used for CZE was 5% (v/v) acetic acid (pH 2.4), while the ESI sheath buffer consisted of 0.2% (v/v) formic acid and 10% (v/v) methanol. Each brain sample was measured in technical triplicate.

MS analysis was performed using the Orbitrap Exploris 480 mass spectrometer in data-dependent acquisition (DDA) mode. Full MS (MS1) scans were acquired at a resolution of 120,000 (at m/z 200), with a single microscan and a scan range of m/z 600–2000. The automatic gain control (AGC) target was set to 300%, and the injection time was automatically adjusted. Precursors were isolated using a 2 m/z isolation window and fragmented by higher-energy collisional dissociation (HCD) at a normalized collision energy (NCE) of 25%. Only precursor ions with intensities greater than 10,000 and charge states between 5+ and 60+ were selected for fragmentation. MS/MS spectra were acquired at a resolution of 60,000 (m/z 200) with three microscans, an AGC target of 100%, and a maximum of six dependent scans per cycle. Dynamic exclusion was enabled for 30 seconds with a 10-ppm mass tolerance, and isotopic peaks were excluded to reduce redundant fragmentation.

Database search for proteoform identification and quantification

Data analysis was performed using Xcalibur software (Thermo Fisher Scientific, version 4.5) to extract proteoform intensities and migration times. Electropherograms were exported from Xcalibur and further processed in Inkscape (version 1.3.2) to generate the final figures.

Proteoform identification and quantification from MS RAW files of zebrafish brain regions were conducted using the TopPIC pipeline (Top-down mass spectrometry-based Proteoform Identification and Characterization)[5]. Initially, RAW files were converted to mzML format using the MSConvert tool [6] . Monoisotopic masses and proteoform features

were extracted from precursor and fragment isotope clusters using TopFD (Top-down mass spectrometry Feature Detection, version 1.7.8)[7]. The resulting mass spectrum and feature data were saved in .msalign and text formats, respectively. The database search was conducted via TopPIC (version 1.7.8), allowing for a maximum of one unexpected mass shift per proteoform. The precursor and fragment mass error tolerances were set to 10 ppm, and unidentified mass shifts were limited to a maximum of 500 Da. A target-decoy approach was applied to estimate false discovery rates (FDRs), and identifications were filtered at 1% FDR at the proteoform-spectrum match (PrSM) level and 5% FDR at the proteoform level [8,9]. A comprehensive list of all identified proteoforms from CZE-MS/MS experiments is provided in **Supporting Information II**.

Label-free quantification of the identified proteoforms was performed using TopDiff (Top-down mass spectrometry-based identification of Differentially expressed proteoforms, version 1.7.8) under default parameters [10]. The analysis was conducted using the software's default parameters. The TopDiff software is part of the TopPIC suite package (https://www.toppic.org/software/toppic/index.html). To determine the differentially expressed proteoforms between zebrafish brain regions (Tele, combined Tec/Hab, Cer, and Med), the quantified proteoforms were further analyzed by the Perseus software[11] . Data preprocessing in Perseus was performed using the following criteria to define quantified proteoforms for pairwise comparisons between brain regions: proteoforms were required to have valid intensity values in at least two of the three technical replicates in at least one brain region. Missing intensity values were handled by imputation using a normal distribution–based approach implemented in Perseus. The resulting dataset of quantified proteoforms was then subjected to Student's t-test analysis in Perseus to identify proteoforms with statistically significant differences in abundance between brain regions. Moreover, we performed PCA using two complementary approaches. First, we uploaded the full proteoform-intensity data set, including all brain regions and their technical replicates, into Perseus. For this dataset, we applied log2 transformation and imputed missing values using the "replace missing values from normal distribution" method before conducting PCA on the complete matrix. In the second approach, we followed the same preprocessing steps but then performed multiple-sample statistical testing to identify differentially expressed proteoforms. PCA was subsequently carried out only on these statistically regulated proteoforms to examine region-specific separation more clearly.

Quantitative bottom-up proteomics (BUP) of zebrafish brain regions

Aliquots of zebrafish brain region lysates, prepared as described in the "Sample Preparation" section, underwent buffer exchange, and the desalted proteins were reconstituted in 30–40 µL of 10 mM ammonium acetate (pH 6.9). Protein reduction was carried out by adding varying amounts of DTT depending on the brain region. For example, 26 µL of 10 mM DTT was added to the combined Tec/Hab sample, followed by

incubation at 37 °C for 30 minutes. Alkylation was then performed by adding 50 µL of 10 mM IAA and incubating the mixture in the dark at room temperature for 20 minutes. The alkylation reaction was quenched by adding 2 µL of 100 mM DTT. Next, 26 µL of trypsin solution (0.1 µg/µL) was added to the same sample for enzymatic digestion, which was carried out overnight at 37 °C. Digestion was terminated by acidifying the sample with acetic acid. The digests were subsequently lyophilized using a vacuum concentrator (Thermo Fisher Scientific) and stored at −80 °C until further analysis. The same protocol was applied to other brain regions, including the Cer, Tele, and Med. The resulting protein digests were then subjected to CZE-ESI-MS/MS in technical triplicate. To do the database search for BUP, CZE–MS/MS raw files were processed in MaxQuant (version 2.6.7.0) using the Andromeda search engine[12]. The Danio rerio proteome (ID: UP000000437, 46,582 entries, July,01 2024) downloaded from UniProt (http://www.uniprot.org/) was used for database search. The peptide mass tolerances of the first and main searches were 20 and 4.5 ppm, respectively. The fragment ion mass tolerance was 20 ppm. Trypsin was selected as the protease. Oxidation on methionine, protein N-terminal acetylation, and phosphorylation on serine, threonine, and tyrosine were specified as variable modifications, while carbamidomethyl on cysteine was defined as a fixed modification. The minimum peptide length was set to seven amino acids, and both peptide- and protein-level FDRs were controlled at 1%.

Table S1. Mass, estimated volume, and lysis buffer volume of dissected zebrafish brain regions.

**Brain Region**	**Avg Mass(mg) Single brain**	**Estimated Volume(mm^3) Single brain**	***Mass (mg) five brains***	***Lysis buffer volume (μL)***
**Medulla**	2.1	0.5	10.5	250
**Cerebellum**	4.0	1.0	20	440
**Tectum+Habenula**	8.3	2.0	41.5	1000
**Telencephalon**	3.5	0.8	17.5	360

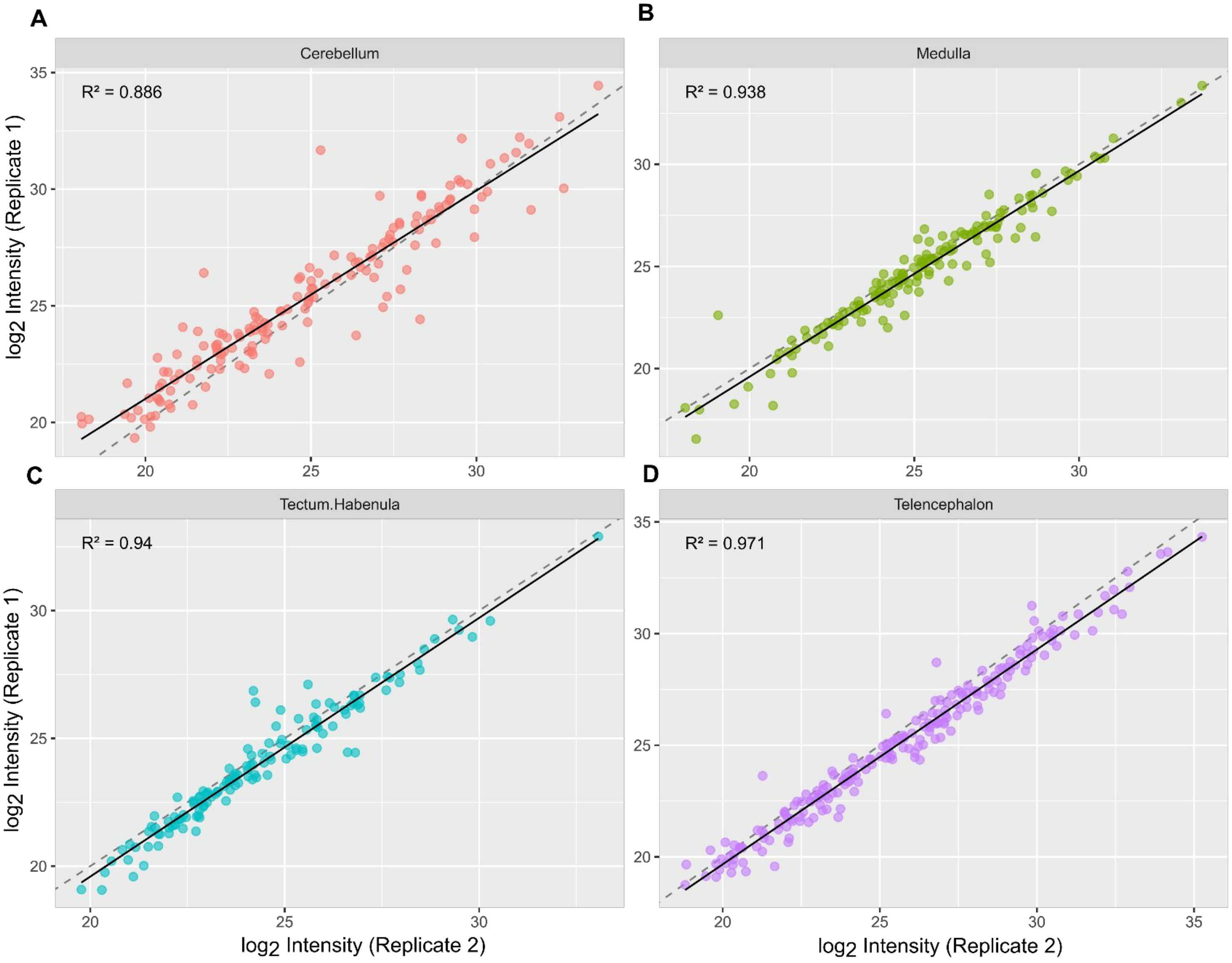

Figure S1. Reproducibility assessment of proteoform intensity between technical replicates of CZE-MS. Log_2-transformed proteoform intensity values were used here. (A) Cer, (B) Med, (C) Combined Tec/Hab, and (D) Tele. Each point represents a quantified proteoform, and the solid black line indicates the linear regression fit, and the gray dashed line represents the ideal 1:1 line reflecting perfect agreement between replicates.

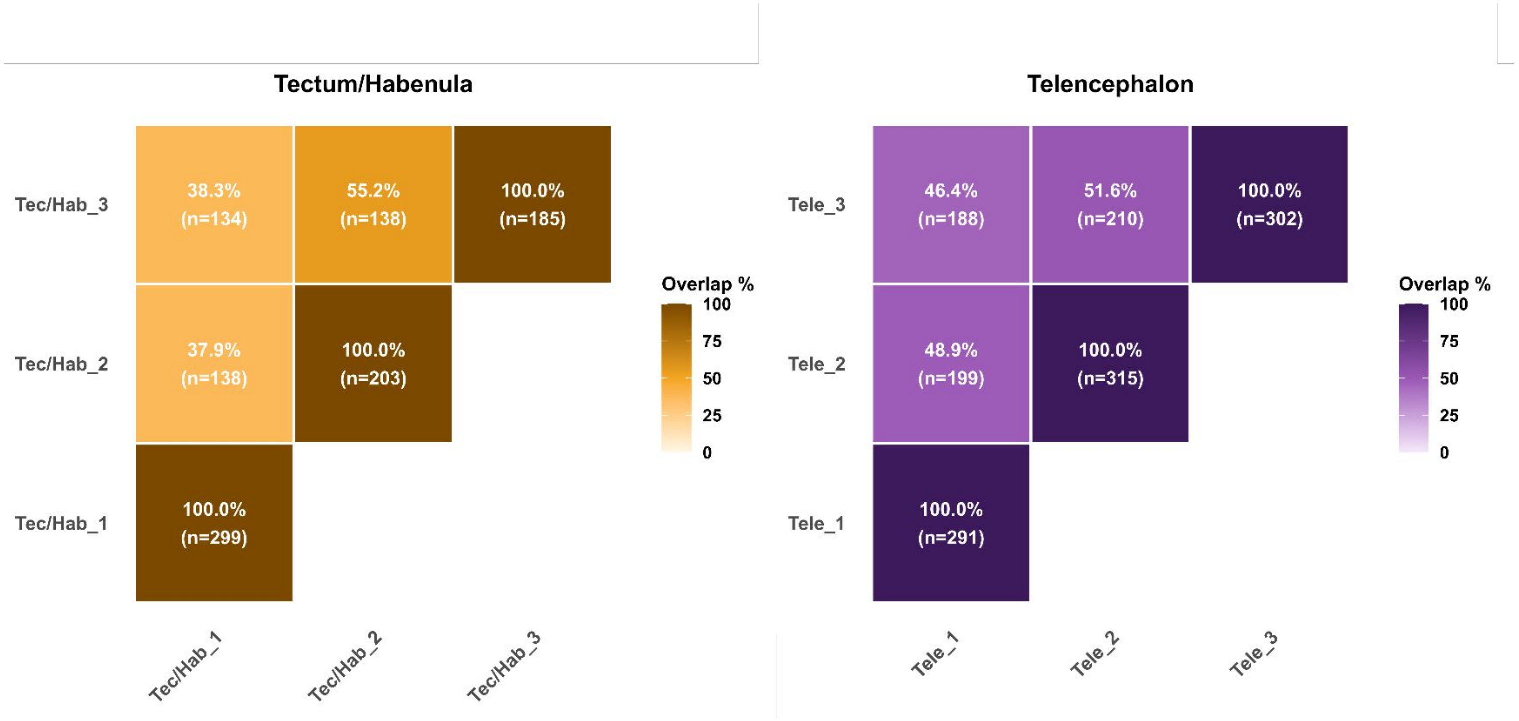

Figure S2. Proteoform overlaps between replicates. Pairwise overlap heatmaps for Tec/Hab (orange) and Tele (purple) replicates, showing the percentage and number of shared proteoforms between each replicate pair.

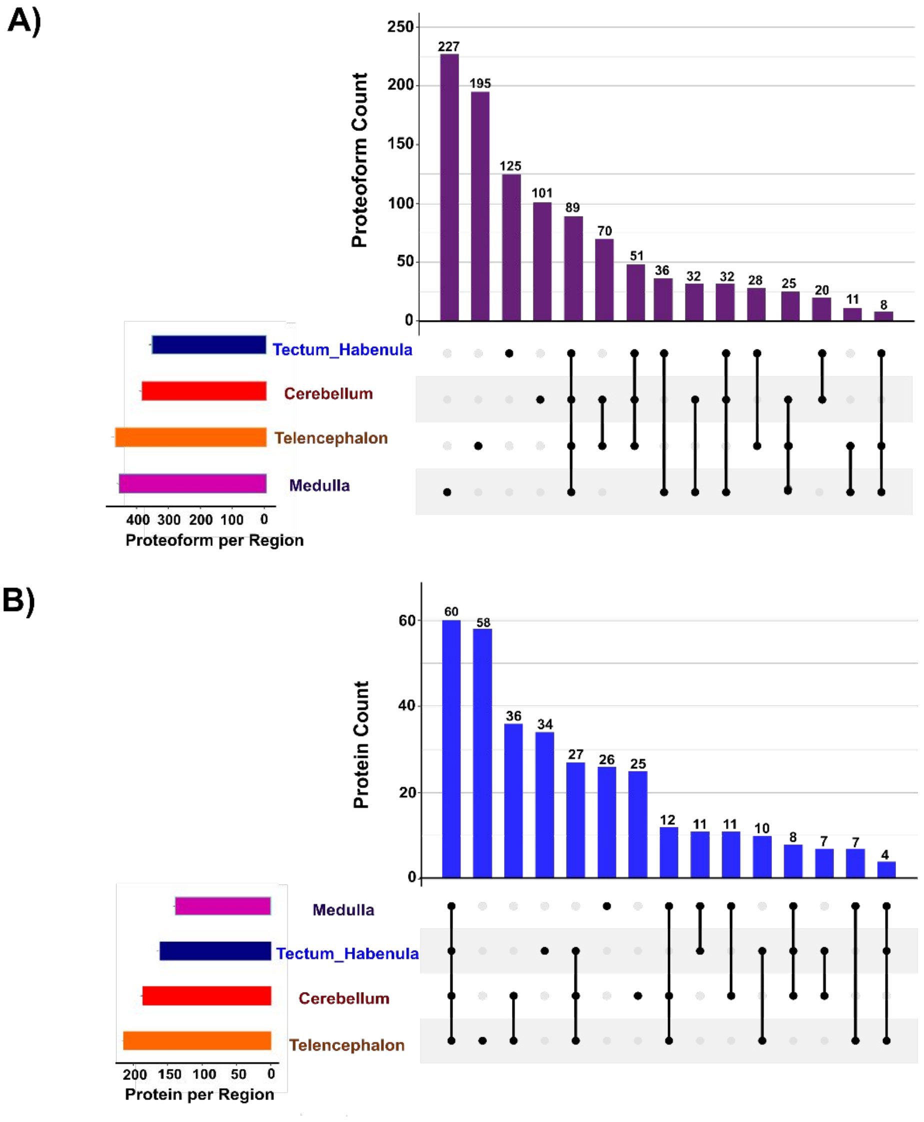

Figure S3. UpSet plots summarize the distribution of proteoforms and proteins across zebrafish brain regions. (A) Proteoform-level comparison among Cer (420), Med (460), Tele (477), and Combined Tec/Hab (389), yielding a total of 1,050 proteoforms. A subset of 89 proteoforms (5.1%) was shared across all four regions, while the majority were region-specific, with Tele contributing the largest proportion (27.3%). (B) Protein-level comparison across the same regions. A total of 336 proteins were identified, of which 60 (17.9%) were shared among all regions. Unique proteins were most abundant in the Tele (58, 17.3%), followed by Combined Tec/Hab (34, 10.1%), Med (26, 7.7%), and Cer (25, 7.4%), with additional proteins distributed across pairwise and triplet overlaps.

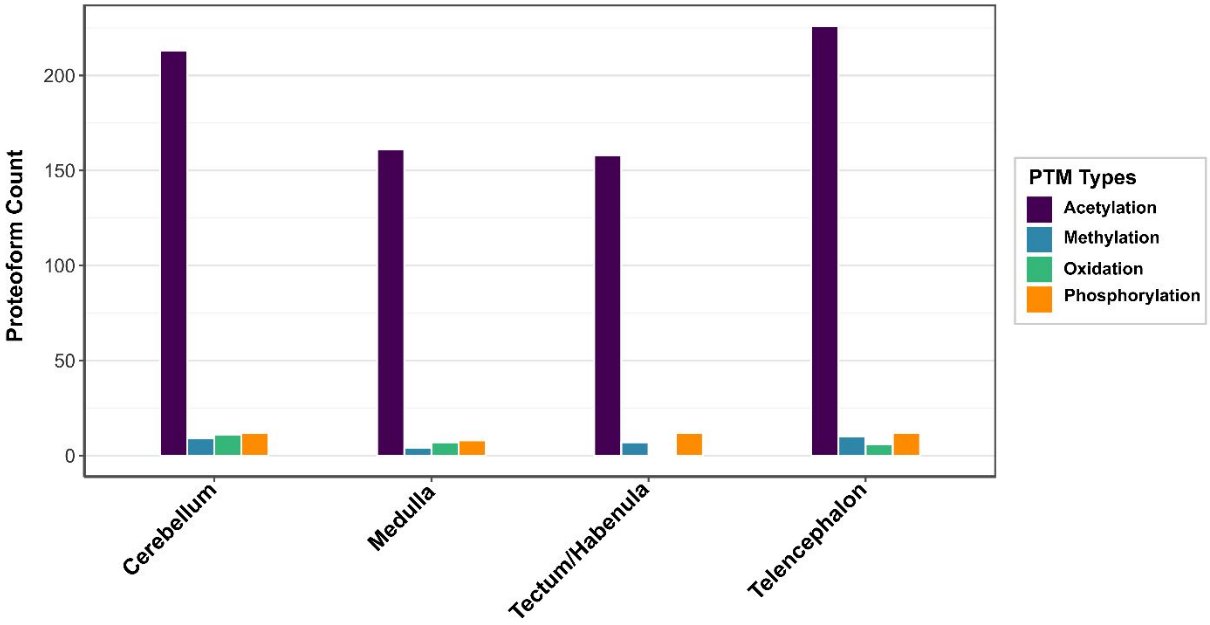

Figure S4. Distributions of proteoforms carrying common post-translational modifications (PTMs, e.g., acetylation, phosphorylation, methylation, and oxidation) were identified in the four brain regions (Tele, Tec/Hab, Cer, and Med).

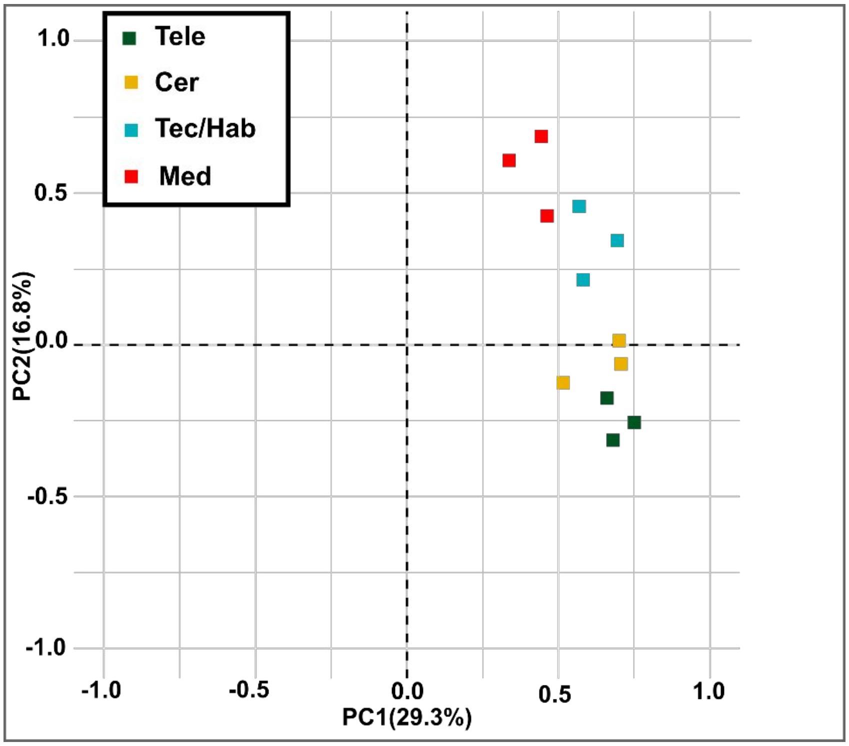

Figure S5. Principal component analysis of proteoform intensity profiles across zebrafish brain regions. PCA plot displays separation of Tele (green), Cer (yellow), Tec/Hab (cyan), and Med (red) based on whole proteoform intensity data. PC1 and PC2 account for 29.3% and 16.8% of variance, respectively.

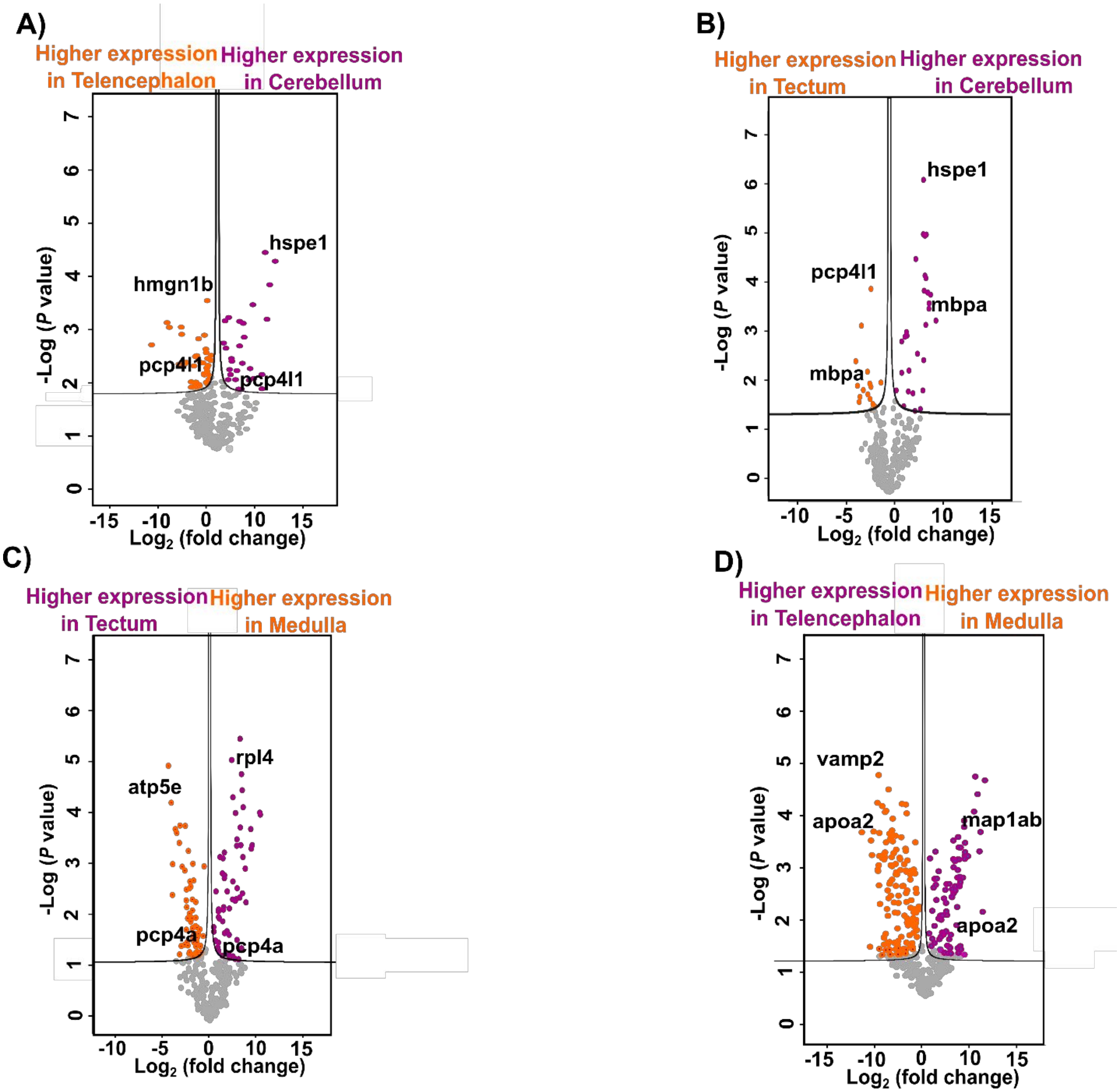

Figure S6. Volcano plot analysis of differentially expressed proteoforms across the zebrafish brain region. (A) Tele vs. Cer comparison showing higher abundance proteoforms in Tele (orange) and Cer (purple). (B) Tec/Hab vs. Cer comparison displaying proteoforms with elevated expression in Tec/Hab (orange) and Cer (purple). (C) Tec/Hab vs. Med comparison illustrating differential proteoforms in Tec/Hab (purple) and Med (orange). (D) Tele vs. Med comparison revealing proteoforms with higher abundance in Tele (purple) and Med (orange). Selected differential proteoforms are labeled with their corresponding gene names. Volcano plots were generated using Perseus software with parameters set to S0 = 0.1 and FDR = 0.1. Grey dots represent non-significant proteoforms.

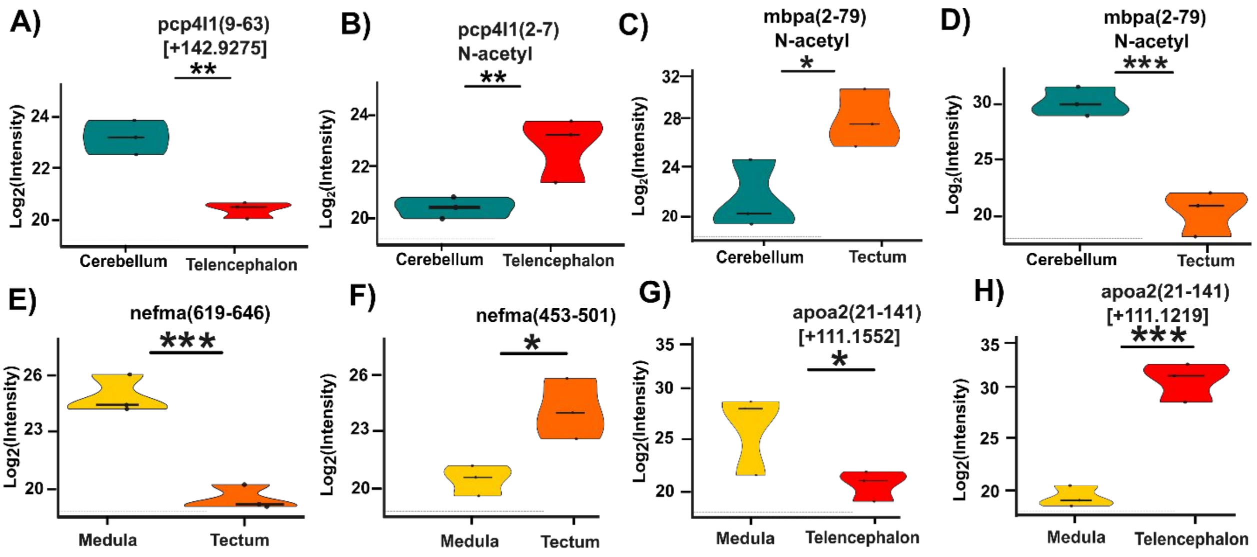

Figure S7. Violin plots showing bidirectional proteoform regulation across zebrafish brain regions. Abundance differences of specific proteoforms between brain regions: (A) pcp4l1(9-63) proteoform between Cer and Tele, (B) pcp4l1(2-7) N-acetyl proteoform between Cer and Tele, (C) mbpa(2-79) N-acetyl proteoform between Cer and Tec/Hab, (D) mbpa(2-79) N-acetyl proteoform between Cer and Tec/Hab, (E) nefma(619-646) proteoform between Med and Tec/Hab, (F) nefma(453-501) proteoform between Med and Tec/Hab, (G) apoa2(21-141) proteoform between Med and Tele, and (H) apoa2(21-141) proteoform between Med and Tele. Mass shifts are indicated in brackets. Statistical significance: * $p < 0.05$, ** $p < 0.01$, *** $p < 0.001$.

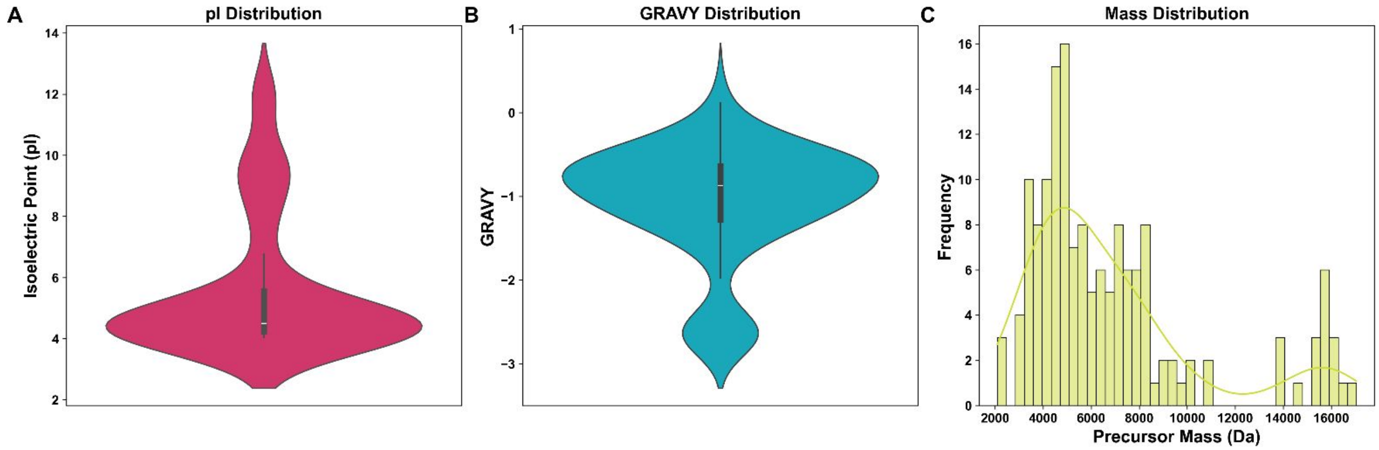

Figure S8. Distribution of physicochemical properties of predicted neuropeptides, including (A) isoelectric point (pI), (B) hydrophobicity (GRAVY score), and (C) Precursor mass. These profiles illustrate the expected biochemical characteristics and diversity of neuropeptide candidates.

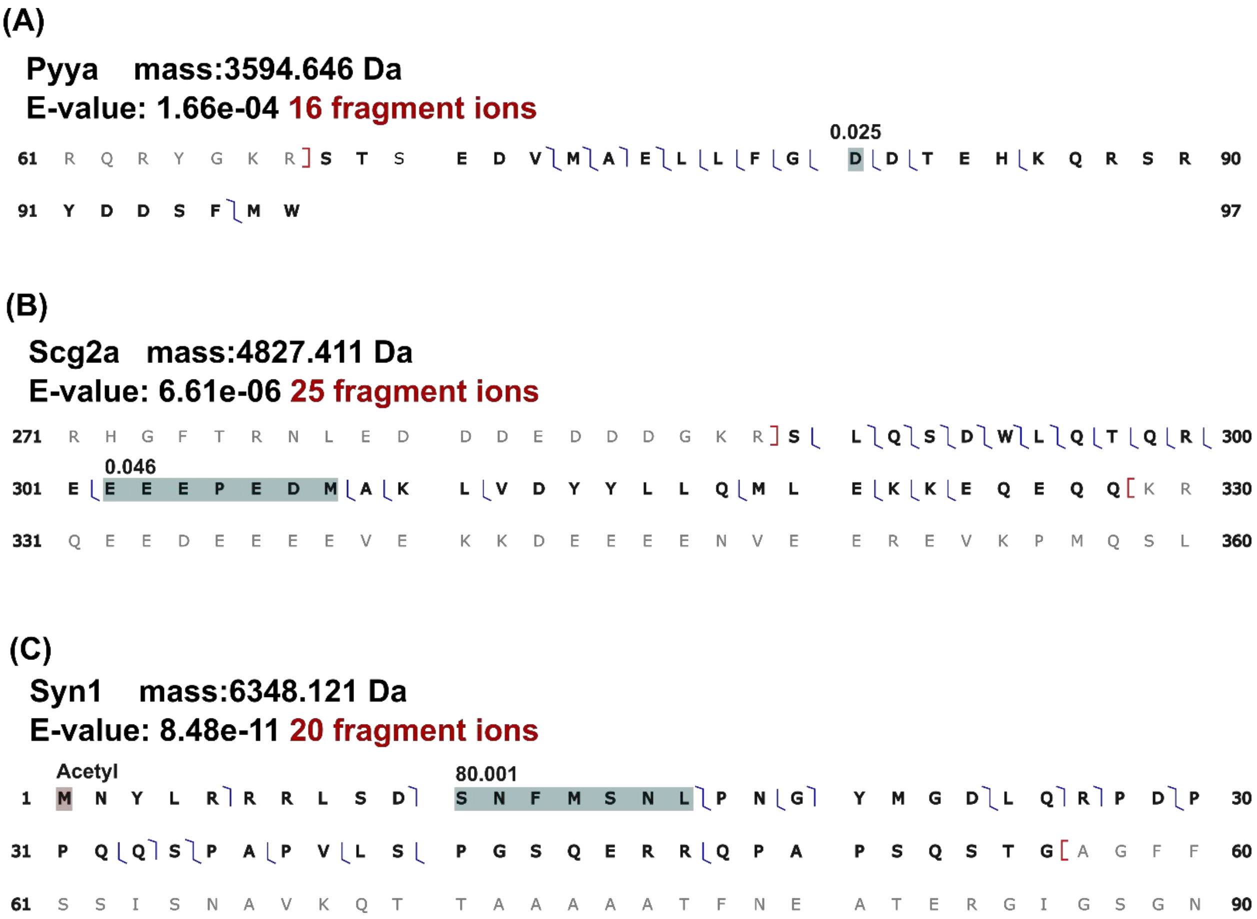

Figure S9. Amino acid sequences and MS/MS fragmentation patterns of three identified proteoforms. A) Pyya truncated proteoform. B) Scg2a truncated proteoforms. C) Syn1 N-terminally acetylated proteoform with a mass shift of +80 Da. Marked amino acid residue regions indicate potential modification sites. Exact modification sites cannot be determined in most cases due to limited backbone cleavage coverage.

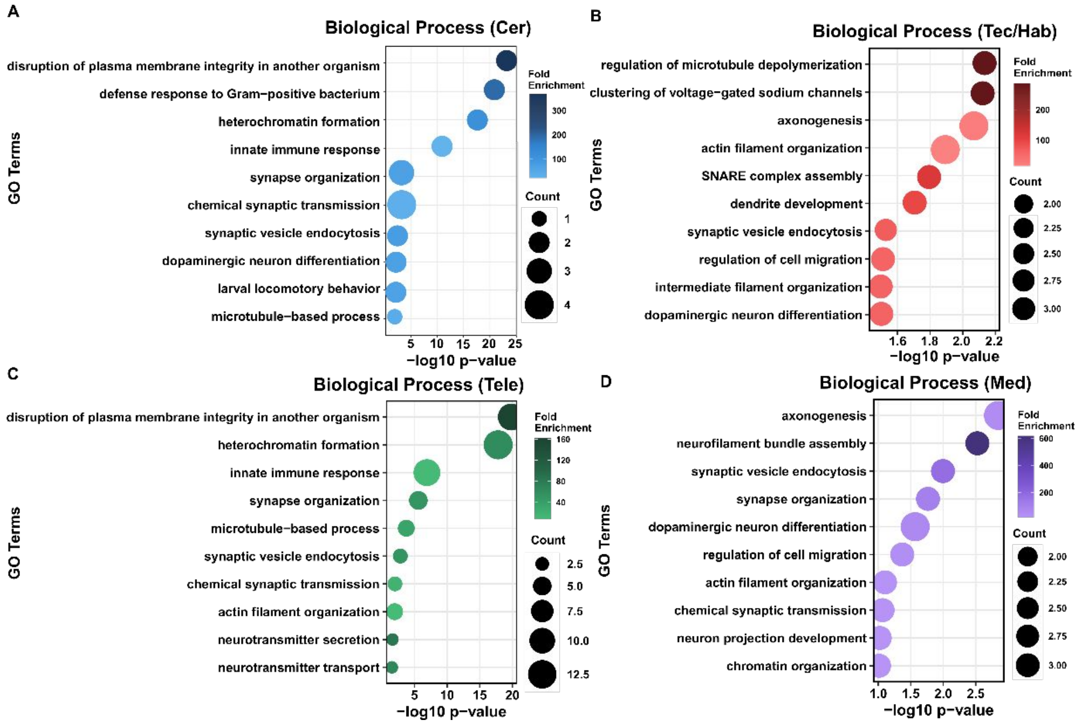

Figure S10. Gene Ontology (GO) enrichment analysis of genes corresponding to differentially expressed proteoforms in zebrafish Brain regions **(A)** Cer, **(B)** Tec/Hab, **(C)** Tele, **(D)** Med. The X-axis represents the -$\log_{10}$(p-value), indicating the significance of enrichment, while the Y-axis lists the specific Biological Processes. Bubble size corresponds to the number of genes involved, and the color gradient reflects the fold enrichment, with higher values indicating stronger enrichment.

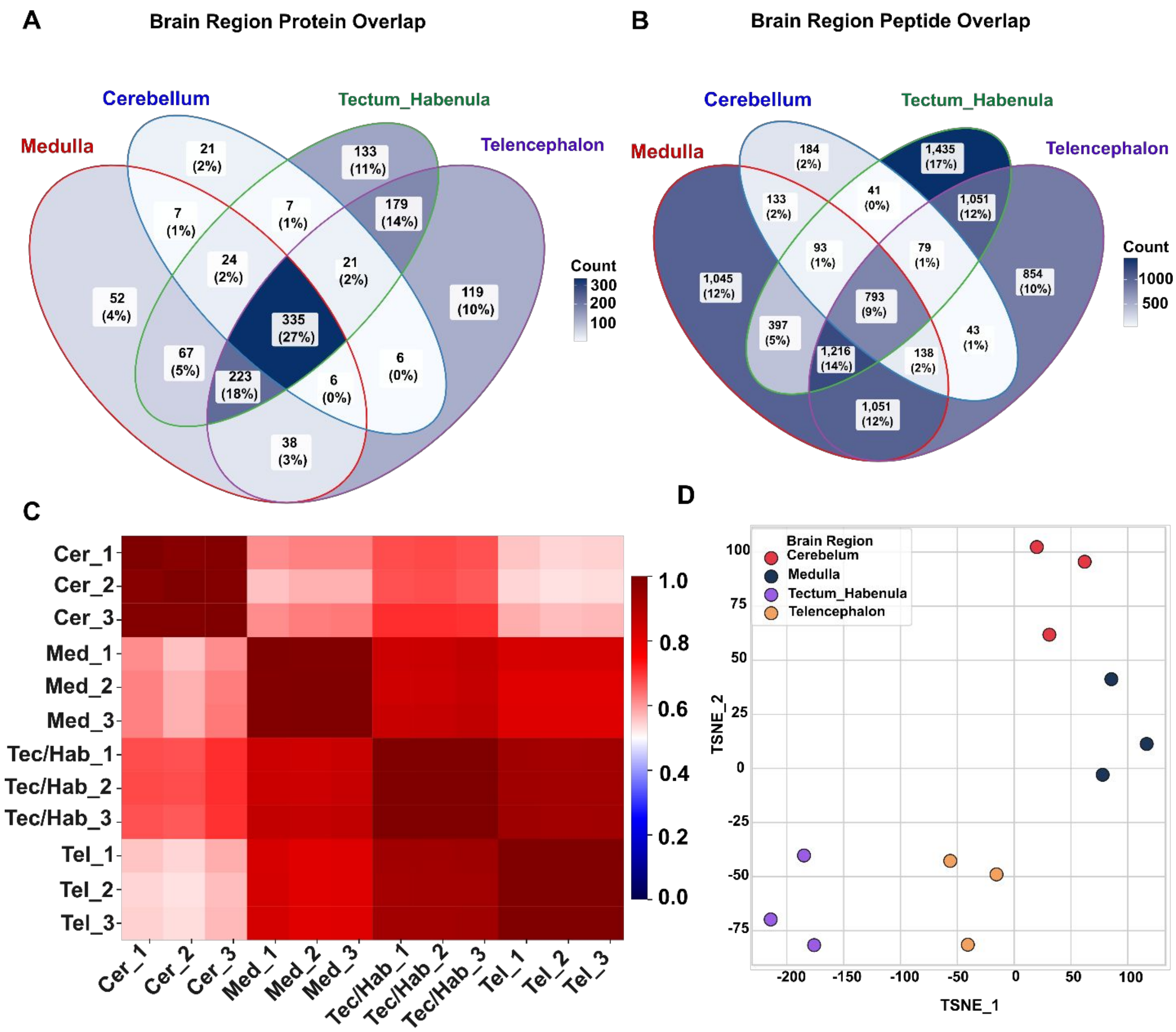

Figure S11. Summary of protein groups and peptides from BUP analysis of different brain regions. (A) Venn diagram showing protein overlap across four brain regions, with 335 proteins (27%) common to all regions. (B) Peptide overlaps with 793 peptides (9%) shared among all regions. (C) Correlation heatmap of protein group intensities between technical replicates (Cer, Med, Tec/Hab, Tele; _1, _2, _3 indicate replicates). (D) t-SNE visualization showing distinct clustering of brain regions based on proteomic profiles.

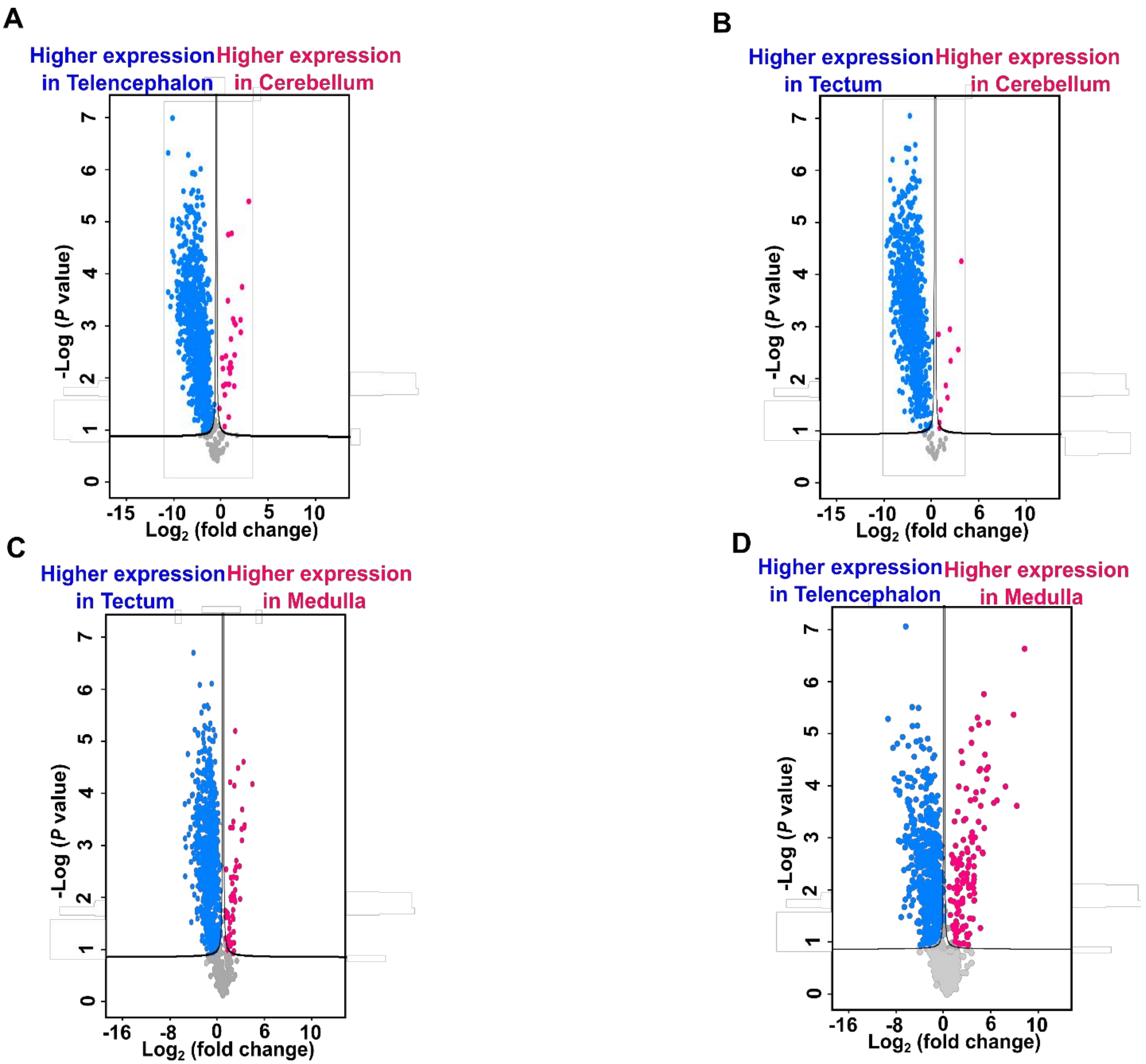

Figure S12. Volcano plots showing the differentially expressed protein groups from quantitative bottom-up proteomics (BUP). Volcano plots display protein group quantification from BUP analysis comparing (A) Tele versus Cer and (B) Tec/Hab versus Cer, (C) Tec/Hab vs Med, and (D) Tele versus Med regions in zebrafish brains, with statistical significance determined using t-test, parameters of false discovery rate (FDR) = 0.05 and S0 = 0.1.

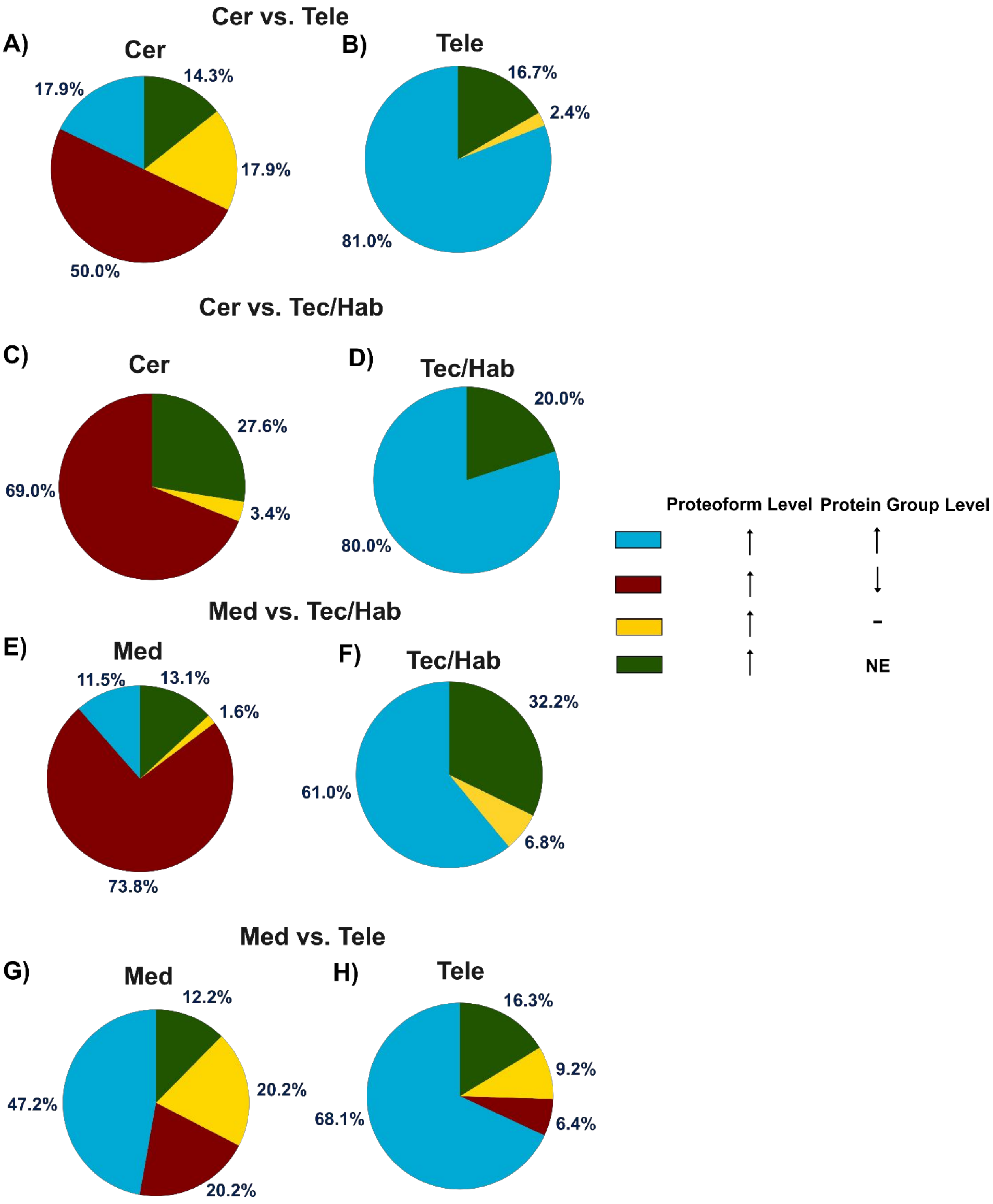

Figure S13. Comparative analysis of quantitative BUP and TDP data for comprehensive assessment of gene expression products at protein group-level and proteoform levels. Pie charts illustrating concordance between TDP and BUP quantitative data for differentially expressed proteoforms across zebrafish brain region comparisons: (A) Cer

and (B) Tele show proteoforms differentially expressed in the Cer vs. Tele comparison, highlighting those enriched in Cer and Tele, respectively. (C) Cer and (D) Tec/Hab show proteoforms differentially expressed in the Cer vs. Tec/Hab comparison, highlighting those enriched in Cer and Tec/Hab, respectively. (E) Med and (F) Tec/Hab show proteoforms differentially expressed in the Med vs. Tec/Hab comparison, highlighting those enriched in Med and Tec/Hab, respectively. (G) Med and (H) Tele show proteoforms differentially expressed in the Med vs. Tele comparison, highlighting those enriched in Med and Tele, respectively. Color coding represents expression patterns at proteoform vs protein group levels. "NE" indicates proteins not detected; "-" denotes no statistically significant differential expression.

References

(1) Sun, L.; Zhu, G.; Zhang, Z.; Mou, S.; Dovichi, N. J. Third-Generation Electrokinetically Pumped Sheath-Flow Nanospray Interface with Improved Stability and Sensitivity for Automated Capillary Zone Electrophoresis-Mass Spectrometry Analysis of Complex Proteome Digests. *J Proteome Res* **2015**, *14* (5), 2312–2321. https://doi.org/10.1021/acs.jproteome.5b00100.

(2) Wojcik, R.; Dada, O. O.; Sadilek, M.; Dovichi, N. J. Simplified Capillary Electrophoresis Nanospray Sheath-Flow Interface for High Efficiency and Sensitive Peptide Analysis. *Rapid Communications in Mass Spectrometry* **2010**, *24* (17), 2554–2560. https://doi.org/10.1002/rcm.4672.

(3) McCool, E. N.; Lubeckyj, R.; Shen, X.; Kou, Q.; Liu, X.; Sun, L. Large-Scale Top-down Proteomics Using Capillary Zone Electrophoresis Tandem Mass Spectrometry. *J Vis Exp* **2018**, No. 140. https://doi.org/10.3791/58644.

(4) Sun, L.; Zhu, G.; Zhao, Y.; Yan, X.; Mou, S.; Dovichi, N. J. Ultrasensitive and Fast Bottom-up Analysis of Femtogram Amounts of Complex Proteome Digests. *Angewandte Chemie - International Edition* **2013**, *52* (51), 13661–13664. https://doi.org/10.1002/anie.201308139.

(5) Kou, Q.; Xun, L.; Liu, X. TopPIC: A Software Tool for Top-down Mass Spectrometry-Based Proteoform Identification and Characterization. *Bioinformatics* **2016**, *32* (22), 3495–3497. https://doi.org/10.1093/bioinformatics/btw398.

(6) Kessner, D.; Chambers, M.; Burke, R.; Agus, D.; Mallick, P. ProteoWizard: Open Source Software for Rapid Proteomics Tools Development. *Bioinformatics* **2008**, *24* (21), 2534–2536. https://doi.org/10.1093/bioinformatics/btn323.

(7) Basharat, A. R.; Zang, Y.; Sun, L.; Liu, X. TopFD: A Proteoform Feature Detection Tool for Top-Down Proteomics. *Anal Chem* **2023**, *95* (21), 8189–8196. https://doi.org/10.1021/acs.analchem.2c05244.

(8) Elias, J. E.; Gygi, S. P. Target-Decoy Search Strategy for Increased Confidence in Large-Scale Protein Identifications by Mass Spectrometry. *Nat Methods* **2007**, *4* (3), 207–214. https://doi.org/10.1038/nmeth1019.

(9) Keller, A.; Nesvizhskii, A. I.; Kolker, E.; Aebersold, R. Empirical Statistical Model to Estimate the Accuracy of Peptide Identifications Made by MS/MS and Database Search. *Anal Chem* **2002**, *74* (20), 5383–5392. https://doi.org/10.1021/ac025747h.

(10) Lubeckyj, R. A.; McCool, E. N.; Shen, X.; Kou, Q.; Liu, X.; Sun, L. Single-Shot Top-Down Proteomics with Capillary Zone Electrophoresis-Electrospray Ionization-Tandem Mass Spectrometry for Identification of Nearly 600 Escherichia Coli Proteoforms. *Anal Chem* **2017**, *89* (22), 12059–12067. https://doi.org/10.1021/acs.analchem.7b02532.

(11) Tyanova, S.; Temu, T.; Sinitcyn, P.; Carlson, A.; Hein, M. Y.; Geiger, T.; Mann, M.; Cox, J. The Perseus Computational Platform for Comprehensive Analysis of (Prote)Omics Data. *Nature Methods*. Nature Publishing Group August 30, 2016, pp 731–740. https://doi.org/10.1038/nmeth.3901.

(12) Cox, J.; Mann, M. MaxQuant Enables High Peptide Identification Rates, Individualized Ppb-Range Mass Accuracies and Proteome-Wide Protein Quantification. *Nat Biotechnol* **2008**, *26* (12), 1367–1372.
